# Autoclaved and Extruded Legumes as a Source of Bioactive Phytochemicals: A Review

**DOI:** 10.3390/foods10020379

**Published:** 2021-02-09

**Authors:** Mercedes M. Pedrosa, Eva Guillamón, Claudia Arribas

**Affiliations:** 1 Food Technology Department, SGIT-INIA, Ctra de La Coruña, Km 7.5, 28040 Madrid, Spain; arribas.claudia@inia.es; 2 Centre for the Food Quality, INIA, C/Universidad s/n, 42004 Soria, Spain; guillamon.eva@inia.es

**Keywords:** pulses, autoclaving, extrusion, galactosides, phytates, protease inhibitors, phenols

## Abstract

Legumes have been consumed since ancient times all over the world due to their easy cultivation and availability as a low-cost food. Nowadays, it is well known that pulses are also a good source of bioactive phytochemicals that play an important role in the health and well-being of humans. Pulses are mainly consumed after processing to soften cotyledons and to improve their nutritive and sensorial characteristics. However, processing affects not only their nutritive constituents, but also their bioactive compounds. The final content of phytochemicals depends on the pulse type and variety, the processing method and their parameters (mainly temperature and time), the food matrix structure and the chemical nature of each phytochemical. This review focuses on the changes produced in the bioactive-compound content of pulses processed by a traditional processing method like cooking (with or without pressure) or by an industrial processing technique like extrusion, which is widely used in the food industry to develop new food products with pulse flours as ingredients. In particular, the effect of processing methods on inositol phosphates, galactosides, protease inhibitors and phenolic-compound content is highlighted in order to ascertain their content in processed pulses or pulse-based products as a source of healthy phytochemicals.

## 1. Introduction

Pulses, which are the dry seeds, separated from their pod, of the Leguminosae family, have been cultivated and consumed for centuries as a staple food for humans. The most common pulses consumed by humans around the world are peas, lentils, beans, soybeans, faba beans, lupins, cowpeas and mung beans [1,2,3]. From a nutritional point of view, they are highly valuable foods since they are high in protein (17–50%), slow-digestion carbohydrates (0.4–55%) and dietary fiber (3–15%), and low in fat (0.8–6.6%) (except the oil seeds: soybeans, peanuts and some lupins) [1,2,3]. Their proteins complement those of cereals well and are gluten-free, being a food of choice for celiacs; furthermore, the demand for pulses has increased recently since more people such as vegetarians, vegans or flexitarians are looking for alternative sources of animal proteins [4,5]. Pulses are cheap, easy to prepare, versatile and in many cases, non-perishable [6]. The growing interest in pulses as both nutritious and healthy foods, led the WHO/FAO to declare 2016 as the “International Year of Pulses” [7], with its main objective to raise awareness of the multiple varieties and benefits of pulses for food security, nutrition, health and environment, and to encourage consumption. In addition, the dietary guidelines of many countries [8] recommend the consumption of 2.5–3.5 cups of pulses per week as part of a healthy diet.

In addition to nutritive compounds, pulses contain numerous phytochemicals, traditionally considered as anti-nutritional factors, and in recent decades as non-nutritional components; although most of them are non-toxic for humans, they can produce some discomfort (e.g., flatulence) and interfere with protein digestibility and the bioavailability of some nutrients such as minerals [3,6,9]. However, nowadays these phytochemicals are well recognized as bioactive compounds able to exert a beneficial/healthy effect when ingested on a regular basis, reducing the incidence of several chronic diseases such as type 2 diabetes, cardiovascular diseases or some types of cancer; thus, pulses are recognized as functional foods [6,9]. These health effects may be associated with more than one bioactive compound, and synergistic combinations may exist [3,6,9,10]. These bioactive substances are not equally present in all legumes (seeds and varieties); for example, the common bean shows the highest levels of lectins, while soybean is rich in trypsin inhibitors, and peas contain higher amounts of α-galactosides [9,11]. According to different authors [3,9], depending on the compound, its concentration in the food, the time at which it is consumed and its interaction with other food matrix components, these compounds can act as anti-nutrients or as bioactive compounds Therefore, depending on the compound, it may be desirable to reduce or increase its content but not remove it completely from food products [3,9,11]. It is noteworthy that processing of pulses or pulse-based mixtures can also increase, reduce/inactivate or produce minor changes in the content of other non-nutritive components such as protease inhibitors, galactosides, lectins, phenols or phytates [1,5,12,13,14,15,16]. Although the beneficial effects of these components mainly depend on their bioavailability in the gut, and since there currently is not a recommended daily intake of bioactive compounds, it would be of great interest to know the level of each bioactive compound to be consumed in the processed foods, because processing can disrupt the food matrix, making phytochemicals more or less bioaccessible [17].

There are a number of processing techniques available, such as soaking, dehulling, germination, malting, fermentation, cooking, autoclaving, microwaving, roasting or extrusion, that make it possible to achieve the suitable nutritional and organoleptic characteristics of pulses, as well as to improve the content of bioactive compounds in comparison with the raw products. Different processing methods can affect, to a different extent, the content of a specific compound. Soaking is very effective in reducing water-soluble compounds such as oligosaccharides and some phenolic compounds. Germination reduces phytic acid effectively, while cooking is more effective in reducing bioactive compounds that are heat-labile, such as protease inhibitors and lectins [9]. In relation to the phytochemical content of the processed pulses, there is a great variability in the literature on the data for the same processing method [5,9,12,18,19,20]. For example, for a specific compound, some authors report contradictory findings. In general, the final effect of a processing method depends on the pulse type and variety, the processing parameters (mainly amount of water, temperature and time), the food matrix structure, the chemical nature of each phytochemical and the presence of additional compounds that may protect each other during processing [3,9,11,21].

Although different review papers can be found in the literature on the content of several bioactive compounds in pulses [9,10,11,22,23], reviews focused on the bioactive-compound content in processed pulses or in pulse-based foods are scarce, and their content is more limited than the present review, since some of them are concerned only with one type of treatment (for example, boiling), or in one type of bioactive compound (mainly phenolic compounds), and to the best of our knowledge, there are no reviews about the cold extrusion process [11,17,21,24].

This review is focused on the changes produced in the bioactive phytochemical content of pulses processed either by a traditional processing method such as cooking with pressure either domestically or industrially, or by industrial processing techniques like extrusion/cooking or cold extrusion, which are widely used in the food industry to develop new food products with pulse flours as ingredients. In particular, the effect of the processing method on some of the bioactive phytochemicals present in pulses (inositol phosphates, galactosides, protease inhibitors and phenolic compounds) is highlighted in order to ascertain the content of these bioactive compounds in processed pulses or pulse-based products as a source of healthy phytochemicals.

## 2. Methodology

The methodology followed in the elaboration of this systematic review first included first the selection of the topics addressed (Figure S1). For this, domestic and/or industrial processing techniques were taken in consideration. Second, the most common bioactive compounds (α-galactosides, myo-inositol phosphates, protease inhibitors and phenolic compounds) present in the legumes usually consumed and related to healthy roles were chosen. With the information obtained about these specific issues, the structure of this review is as follows: (i) content of some bioactive compounds in raw pulses and their health effect in humans; (ii) effect of autoclaving on the bioactive compounds of pulses; (iii) effect of extrusion/cooking on the bioactive compounds of pulses; and (iv) effect of cold extrusion on the bioactive compounds of pulses.

After establishing the subject of study (autoclaved, extruded legumes and bioactive compounds), a search was conducted on different scientific databases: SciELO, Science Citation Index, Science Direct, Google Scholar, Medline and Mendeley, and thanks to cross-references, some papers were found and reviewed. Although no restriction was applied for the publication dates, almost all the records identified (85%) corresponded to papers in the last 20 years, and only 15% of the records corresponded to papers published from 1980 to 2000. For each topic, appropriate keywords were used to search for relevant papers. Terms such as phytochemicals, bioactive compounds, the specific name of the phytochemical (α-galactosides, myo-inositol phosphates, protease inhibitors and phenolic compounds), the names of the specific processing method (autoclaving, extrusion/cooking, cold extrusion), canning, snack, pasta and the names of the most common legumes consumed were used as keywords in all possible combinations during the paper-identification step. The results of this search showed that the effect of processing on bioactive compounds of legumes should be organized by taking into account three main factors: the processing procedure, the type of bioactive compound and type of legume. After the search step, some papers were excluded based on the following criteria: (i) record duplicates, (ii) age (before 2000, except those papers that include relevant information that was not reported in more recent papers), (iii) missing information about the processing conditions, and (iv) no clear information about the effect of the processing, making the comparison to the results of other papers difficult. The quality assessment of data was evaluated, taking into account the accessibility of the publication, studies relevant in the area and cited by many other authors, papers that studied more than one phytochemical and papers that evaluated the processing method effect in interaction with more than one other factor (temperature, time, cultivars or the presence of other materials/ingredients). In the case of papers considering more than one treatment or more than one legume, data were considered separately. Finally, 164 research papers (Figure S1) about the phytochemical content on legumes and comparing the effect of autoclaving, extrusion/cooking and cold extrusion on the main bioactive compounds present in legumes (galactosides, myo-inositol phosphates, protease inhibitors and phenolic compounds) were selected. Among these, 143 papers corresponded to studies published in the last 20 years. Data were summarized in tables reporting, for each processing method and legume or their mixtures, the content of each bioactive compound and the effect of the processing treatment on each compound and the source of the data.

## 3. Content of Some Bioactive Compounds in Raw Pulses and Their Health Effects in Humans

### 3.1. α-Galactosides

The most common oligosaccharides in pulse seeds are α-galactosides or the raffinose family of oligosaccharides (raffinose, stachyose, verbascose and ajugose), raffinose and stachyose being the most ubiquitous sugars. Some authors [3,5,6,9,10,23] have reviewed the content of α-galactosides in different seeds and varieties, ranging from 0.4 to 16.1% (dry matter—d.m). Singh et al. [11] reported that lentils presented the lowest amount of total galactosides (37.5 mg/g), followed by faba beans (52.0 mg/g), beans (60.9 mg/g) and peas (66.3 mg/g). Muzquiz et al. [9] reported concentrations of raffinose, ciceritol, stachyose and verbascose in different varieties of Spanish beans, peas, chickpeas, faba beans, soybeans and lupins; raffinose ranged from 1.0 mg/g in *Phaseolus vulgaris* var. Palmeña to 33.15 mg/g in *Lupinus mariae-josephi*; stachyose varied from 9.22 mg/g in *Vicia faba* var. Alameda to 59.08 mg/g in *Lupinus albus* var Multolupa. Verbascose was not detected in the reported chickpeas, the highest amount being found in *Pisum sativum* var. Luna (50.25 mg/g). Another α-galactoside is ciceritol, an α-D-digalactoside of pinitol that does not belong to the raffinose family of oligosaccharides. It is not present in all pulses, and its amount ranged from 1.61 mg/g to 29.65 mg/g in pea var. Iceberg and chickpea var. Duraton, respectively.

The α-galactosides are not digested or hydrolyzed by humans. However, they are fermented by colonic bacteria with the production of hydrogen, carbon dioxide, methane and short-chain fatty acids (SCFA), mainly butyric and propionic. While the gases produced are responsible for flatulence, bloating and diarrhea, the SCFA are mainly related to the prebiotic effect associated with the α-galactosides, promoting the growth of beneficial gut microflora (bifidobacterias and lactobacilli) and reducing the enterobacteria population. α-Galactosides have also been shown to help in normalizing bowel function, reducing potentially carcinogenic compounds (such as N-nitroso compounds), and enhancing the immune system and increasing resistance to infection [3,9,11,25]. In addition, it has also been reported that propionic acid reduces serum cholesterol, helping to reduce the risk of cardiovascular diseases, and butyric acid induces apoptosis and stops the growth and differentiation in colon cancer cells [3,9,11]. Despite there not being a recommended dietary intake for α-galactosides, Martinez-Villaluenga et al. [25] documented that a dose of 3 g/day of α-galactosides produces an increase in the intestinal bacteroides, bifidobacterias and eubacteria without any flatulence discomfort.

### 3.2. Myo-Inositol Phosphates

Phytate (IP6) or myo-inositol hexakisphosphate is the main form of phosphorous storage in pulse seeds (up to 75% of total seed phosphorous), and is stored as salts (phytate–mineral complex) or bound with proteins or starch [3,9]. According to the literature, the total content of inositol phosphates ranges from 0.2 to 2.34% [3,9,10,11,23], and as described above for α-galactosides, the amount of inositol phosphates varies between species and varieties, as well as with the soil phosphorous [26]. Sparvoli et al. [3] reported that varieties of peas (3.1–7.1 mg/g), chickpeas (2.8–13.6 mg/g), lentils (2.5–12.2 mg/g) and mung beans (1.8–5.8 mg/g) contain relatively lower amounts of IP6 than those of common beans (3.4–28.7 mg/g), faba beans (5.9–15.0 mg/g) and soybeans (4.8–20.1 mg/g). Muzquiz et al. [9] reported the total inositol phosphate content in different varieties of some Spanish legumes, the average content being 0.4% in beans, 0.75% in faba beans, 0.6% in chickpeas and beans, 0.7% in lupins and 1.2% in soybean varieties.

In general, phytate, myo-inositol hexaphosphate or IP6 has been considered as an antinutrient that interferes with nutrient (mineral, protein and starch) digestibility and bioavailability [27]. However, this mechanism of action also produces health benefits. As IP6 binds starch and the calcium necessary for α-amylase activity, the starch digestibility is reduced, improving the glycaemic response of pulses and the management of diabetes type 2 [3,23]. Due to its mineral-binding capacity, IP6 has been linked with other beneficial health effects, such as the prevention of kidney stone formation, the prevention of cavities and plaque in teeth and protection from demineralization. It has been also reported that a diet with 1% sodium phytate added can control hypercholesterolaemia and atherosclerosis, and can reduce the risk of colon cancer and improve irritable bowel syndrome [22,28]. Further, it has been reported that the lower phosphorylated forms (IP5–IP3) can promote the absorption of minerals and show strong antioxidant and anti-inflammatory activities, inducing apoptosis and normalizing abnormal cell proliferation [3,9,22,23]. In addition, some authors [29,30] have reported that iron absorption can be improved when IP6 is below 10 mg/g protein in one serving dose.

### 3.3. Protease Inhibitors

Protease inhibitors in legumes belong to the Kunitz and the Bowman–Birk families, and both are capable of inhibiting trypsin and chymotrypsin enzymes. There is a high number of isoforms of both inhibitors that vary with the legume species and variety [31]. The trypsin inhibitor content ranges from 5.75 to 15 trypsin inhibitor units (TIU)/mg in peas, from 5 to 10 TIU/mg in faba beans, from 12.60 to 19 TIU/mg in chickpeas, from 3 to 8 TIU/mg in lentils and from 8.57 to 83.70 TIU/mg in soybeans [9,19]; while the chymotrypsin inhibitor content varies from 2.19 chymotrypsin inhibitor units (CIU)/mg in *Vicia narbonensis* to 17.30 CIU/mg in bean var. Riñón, but is not detected in soybean var. Ostrumi [9,10]. Protease inhibitors have a negative effect on animal growth due to the inhibition of gut protein digestion. However, in a Western diet, there have not been any reported toxic problems related to the intake of these compounds, mainly because pulses are cooked prior to consumption and protease inhibitors are thermal-labile compounds. Over the past two decades, different studies [3,6,9,22,23,32,33] have shown that protease inhibitors are effective in preventing or reducing colon, lung, liver, prostate and breast cancer progression. Sánchez-Chino et al. [23] reported that some possible mechanisms of action are: (i) the reduction of protein digestibility reduces the availability of essential amino acids for the cancer cells; (ii) the protease inhibitors act as insoluble dietary fiber able to absorb carcinogens (such as free radicals) in the gut; and (iii) the inhibiting of proteases produced by cancer cells. Even though there is not a recommended amount of protease inhibitor consumption, it is important to note that the traditional Japanese diet contains about 420 protease inhibitor units/day; further, it has been reported that the consumption of the purified protease inhibitor at 25–800 CIU per day during a period of 12 weeks exerted a protective effect against cancer development, and doses of up to 2000 CIU/day did not cause health problems in humans [9,10,11,22,32,33,34,35,36].

### 3.4. Phenolic Compounds

Pulses are rich in phenolic compounds and include different subcategories such as tannins, flavonoids, isoflavones, phenolic acids (such as caffeic, ferulic, sinapic and p-coumaric acids) and anthocyanins. Many of these compounds are located in the seed coat and are responsible for seed color, and are related to the taste and flavor of seeds. In general, the darkest legume varieties tend to have higher amounts of phenolic compounds than the light seeds/varieties [3,11,37,38]. Even though a great variability in the phenolic content can be found due to the different methods used in their extraction and quantification (spectrophotometrically or by HPLC (high-performance chromatography)), in general, a high variability can be found among legumes and varieties [5,24,38,39,40,41,42]. For example, among beans, there can be found values from 0.3 mg/g of phenolic compounds in white varieties to 12.6 mg/g in black varieties; peas show values from 0.6 to 2.7 mg/g and chickpeas from 0.6 to 2.7 mg/g [19]. Pedrosa et al. [5] reported that the raw Curruquilla bean, a cream-colored variety, shows a higher content of total phenols and anthocyanins (2.70 mg/g and 40.10 µg/g) than the Almonga bean (2.38 mg/g and 38.47 µg/g), a white variety. Some authors [24,41] reviewed the phenolic content of various legume seeds and documented contents from 11.2 to 48.3 mg/g in dry beans, from 117.8 to 157.6 mg/g in different faba bean genotypes, from 4.9 to 68 mg/g in lentils and from 0.98 to 183 mg/g in chickpeas. Condensed tannins are associated with an astringent taste, as well as with some anti-nutritional effects due to their ability to bind and precipitate proteins, reducing their digestibility. However, from a health point of view, different studies found in the literature report phenolic compounds as bioactive molecules with antioxidant, antimicrobial, anticarcinogenic, immunomodulating, cardio-protective, anti-hypertensive and anti-inflammatory properties, lowering the risk of colon cancer and osteoporosis [6,10,22,38,41]. Most of these actions are linked to the antioxidant and antiradical activities of the different phenolic compounds. In general, antioxidant activity is positively correlated with total phenolic content [41]. Cardador-Martinez et al. [43] reported that white beans possess low antioxidative activity in comparison to black, brown and red bean varieties. As documented in some reviews [11,41], different authors related this high antioxidant activity to pro-anthocyanidins or condensed tannins, as well as to flavonoid content, and concluded that pulses can be a useful natural source of antioxidants. Zhang et al. [44] reported that lentil phenols also inhibit glucosidase and lipase, and would therefore contribute to controlling blood glucose levels and obesity in humans. In spite of these health benefits, there is not in fact a recommended daily intake of phenolic compounds, mainly due to the differences in their total intake for the overall population; however, some reports recommend a minimum daily dose of 300 mg of total phenolic compounds to benefit from their health properties [45].

## 4. Effect of Autoclaving on the Bioactive Compounds of Pulses

Forty-eight papers about the effect of the autoclaving on legume phytochemicals have been consulted for this review. Nine papers evaluated the effect on α-galactosides; 15 papers were about the effect on inositol phosphates content; 18 papers were about the effect on trypsin inhibitor activity and 25 were the effect on phenolics compounds and their antioxidant activity.

### 4.1. Autoclaving Process

Autoclaving (cooking with pressure) is a thermal process usually undertaken at home in a pressure cooker and by the food industry in an autoclave to prepare pulses for human consumption. It is an alternative to boiled legumes, which require cooking in water for very long time. A processing technique such as autoclaving reduces the cooking time and promotes pulse consumption by providing products that are quickly prepared and easy to use.

Autoclaving is a high-pressure cooking technique that uses high temperature and pressure over a short period of time. It is a high-intensity thermal method used in industry to produce tinned legumes commercially. The autoclave is a pressure chamber that operates at 1.8 to 2.0 bar and works by subjecting the item to pressurized saturated steam at 121 °C.

The autoclaving parameters of pressure, temperature and time vary from 15 psi, 116 °C to 127 °C and from 10 to 60 min, respectively. This processing affects the nutritional and antinutritional composition of legumes in different ways. Khatoon et al. [46] reported that pressure cooking of eight whole legumes did not affect the nutrient composition. The mean of in vitro protein digestibility of these pressure-cooked legumes was 79.8%, a higher percentage than for raw materials or even when other cooking techniques were applied, such as microwaving. However, Pedrosa et al. [5] observed significant differences in the nutritional composition of two industrial tinned bean varieties (*P. vulgaris* var. Almonga and var. Curruquilla): increasing protein (>7%) and dietary fiber (>5%); and decreasing fat (>16%), carbohydrate (>15%) and, in general, mineral content (P, Mg, Ca, Fe and Zn) after treatment at 116 °C for 42 min. In autoclaved (121 °C) chickpea for 35 min [47], and faba bean [48] for 30 min, similar effects on nutritional composition were observed, except on protein content, which did not change, nor their amino acid composition. However, in vitro protein digestibility and quality increased with autoclaving [47]. The increment of protein digestibility by autoclaving may be due to the heat-denaturation of protein, and reduction or even abolition of bioactive compounds/anti-nutritional factors such as a trypsin inhibitor, tannins and phytic acid. In addition, autoclaving reduced the B-vitamin content of legumes [46,47,48]. Finally, it should be noted that autoclaving was the most effective in reducing or even abolishing allergenicity in lentil, chickpea and lupin [49,50]. Consumption of tinned beans reduced metabolic risk factors associated with obesity [51], and may be used in a renal patient diet [52]. Autoclaving can also affect the content of the bioactive compounds/anti-nutritional factors present in raw materials, increasing or decreasing their contents, mainly depending on the raw material and the extrusion parameters. Moreover, as has been stated previously in this review, in many cases the elimination of some phytochemicals is not desirable, because small amounts are able to produce beneficial health effects against different chronic diseases.

The effect of autoclaving on the content of the different bioactive phytochemicals reviewed is shown in Table 1.

### 4.2. Autoclaving Effect on the α-Galactosides of Pulses

Autoclaving has been reported to be a more effective heat process in reducing α-galactosides, particularly when processed broths are discarded by leaching them through soaking and also into the canning solutions [54]. The pressure applied during this process forces water into the seeds, increasing the extraction of α-galactosides during cooking [67].

In soaked pinto beans (*P. vulgaris*), autoclaving at 115 °C for 30 min resulted in a more effective heat process in reducing α-galactosides, even with cooking for 90 min. Autoclaving for a shorter time (10 or 20 min) also significantly reduces oligosaccharides (46.9% and 49.4%, respectively), particularly stachyose (50.1% and 51.8%, respectively) [54]. Autoclaving (116 °C, 42 min) of beans var. Almonga and Curruquilla was less effective in reducing α-galactosides (25.11% and 39.42%, respectively), with stachyose again reducing more than raffinose, particularly in Curruquilla (50.26%) [5]. Shimelis and Rakshit [55] found that a combination of soaking (in water or NaHCO_3_) and autoclaving caused a significant reduction in stachyose (75.81–77.72%) and raffinose (64.71–72.41%) contents of var. Roba, var. Awash and var. Beshdesh kidney beans, and the combined effect was significantly higher than autoclaving (lower than 62.5% reduction in raffinose and 71.2% in stachyose) or soaking alone. However, it was necessary to combine sprouting for more than 48 h and autoclaving to abolish or completely eliminate α-galactosides in these varieties of bean seeds. Similar results were found when lentils, previously soaked, were autoclaved at 121 °C for 35 min; raffinose, stachyose and verbascose were significantly reduced, but reductions were mainly observed in raffinose (75%) and verbascose (54%) [64]. The same conditions of processing in chickpea provoked similar reductions in the total α-galactoside content (45.95%), with a 44.14% reduction in raffinose and 42.97% in stachyose, while verbascose was completely eliminated after autoclaving treatment [47]. A more effective heat treatment in the reduction of raffinose (67.44%), stachyose (67.26%) and verbascose (87.40%) in *Dolichos lablab* var. Vulgaris was also found to be autoclaving (121 °C) for 45 min; shorter durations (15 and 30 min) provoked fewer reductions (20.93–63.77%) [61]. Stachyose was significantly decreased in faba beans (21%) through autoclaving for 30 min [48].

In the reviewed studies, without exceptions, autoclaving reduced the α-galactosides content in legumes between 14 and 77% with respect to those of the raw seeds, corresponding to the lowest reduction to autoclaved beans and faba beans and the highest reduction to pinto beans. The content of α-galactosides in the autoclaved legumes ranged from 3.6 mg/g to 30.2 mg/g in different beans, becoming higher when cooking broth was not discarded (60.4 mg/g), 27.7 mg/g in chickpeas, from 23.6 mg/g to 8.50 mg/g in *D. lablab* (depending on processing time: 15–45 min), and around 14 mg/g in faba beans and lentils. The interest in ready-to-eat products such as canned legumes is growing, so, taking into account the remaining α-galactosides content in legumes after this treatment, one serving dose of autoclaved legume (100 g) could supply 0.36–6.04 g for beans (with and without broth), 2,27 g for chickpeas, around 1.4 g for faba bans and lentils and 0.85–2.36 g for *D. lablab*. Therefore, autoclaved legumes will maintain the prebiotic activity due to α-galactosides, according to Villanueva et al. [25].

### 4.3. Autoclaving Effect on the Myo-Inositol Phosphates of Pluses

Autoclaving of legumes affects phytic acid by reducing its content [68,69]. The partial elimination of this phytochemical can contribute to improving digestibility of proteins and starch in legumes, which is mainly observed after this cooking treatment [70]. Autoclaving for 30 min caused significant losses, from 33.72% to 41.61% in phytic acid contents of lentils [64,71] faba beans [48] and chickpeas [47,60,71], and higher losses (59.98–65.93%) have been reported in several bean varieties [5,55,71]. Even autoclaving lima beans for 20 min caused the complete removal of phytic acid content [66].

In addition, the phytate phosphorous contents of the lima bean and five different chickpea cultivars were reduced after autoclaving [60,66]. The increase in total phosphorus after autoclaving (120 °C) of chickpea for 60 min demonstrated that phytic acid was hydrolyzed or decomposed during this processing [60]. Phytic acid (IP6) was the main form that was detected in raw and industrial tinned beans var. Almonga and var. Curruquilla, with about 61% reductions after autoclaving at 116 °C for 42 min, and the contents of less phosphorylated forms (IP5 and IP4) and IP3 were detected only in tinned beans [5]. Similar reductions in phytic acid content were found in other varieties of kidney beans after cooking at 121 °C for 30 min (var. Roba, var. Awash and var. Deshbesh) by Shimelis et al. [55]. Soaking of these beans provoked a significant decrease in their phytic acid content (>17%); however, no differences between the reducing effect of autoclaving without soaking and with previous soaking (in water or NaHCO_3_) were observed. Soaking could contribute to reducing total IP content by leaching during processing and by activation of endogenous phytases; additionally, cooking also contributes by formation of insoluble complexes between phytate and other components [72]. In fact, when the autoclaved bean seeds were analyzed with their corresponding broth, the percentage losses of phytic acid were lower (24.6%) than when the broths were discarded [56]. In contrast, Wang et al. [73] reported that soaking (in water) of six pea (*P. sativum*) varieties did not cause significant reductions in phytic acid content (1.2–1.6%). Autoclaving was more effective in reducing phytic acid content than cooking, but it was necessary to combine autoclaving and sprouting to eliminate phytic acid content. The breakdown of phytate during germination is attributed to the increased activity of the endogenous phytase [55]. Myo-inositol phosphate content in legumes was reduced by autoclaving (2–100%) depending on the legume type and the processing time, except in unsoaked faba beans and pre-soaked green faba beans [62]. Data evaluated in 15 reviewed papers showed a reduction in myo-inositol phosphate content from 53% to 100% for lima beans, from 24% to 66% for beans, from 33% to 46% for chickpeas, from 24% to 46% for lentils, and from 23% to 41% for faba beans. The lowest reductions were found for *D. lablab* (2–8%). Just one reviewed paper showed an increase of myo-inositol phosphate content, from 4 to 10% in unsoaked green and white faba beans, and from 7% to 12% in pre-soaked white bean. Taking into account this effect, one serving dose of autoclaved legumes (100 g) could be beneficial for human health, since they can supply from 8.89 to 1059 mg inositol phosphates/100 g) [3,9,22,23]. Considering a protein content around 20%, the autoclaved legumes can supply from 0.44 to 53 mg inositol phosphates per gram of protein.

### 4.4. Autoclaving Effect on the Protease Inhibitors of Pulses

Trypsin inhibitor activity in legumes was reduced, partially or even completely, by heat treatments, particularly by autoclaving [68,69,74]. The thermostability of the protease inhibitor in legumes depends on legume sources, and also processing conditions such as pH, humidity, time, temperature and pressure [74]. Reactions involving deamidation splitting of covalent bonds, such as hydrolysis of peptide bonds in aspartic acid residues, and interchange or destruction of disulfide bonds, might be involved in the thermal inactivation [75]. Several authors have reported this reduction in trypsin inhibitor activity provoked by autoclaving (with pre-soaking) in chickpeas (83.87%), beans (94–98%) [4,41], lentils (80.87%) [64], faba beans (85%) [48]; and even total inactivation of the trypsin inhibitor in the seeds of beans [55,57,61], lima beans [66] and faba beans [62,76,77]. Different effects on the trypsin inhibitor activity by soaking of legumes can be found in the literature, from significant reduction in soaked kidney beans (6–17%) [55] to even an increase (3.20–19.30%) in soaked peas [73]. Independent of this fact, the elimination effect of autoclaving on the trypsin inhibitor activity did not depend on a previous soaking treatment of kidney beans [55]. Luo and Xie [62] studied the effect of autoclaving combined with soaking and/or dehulling on green and white faba beans. Autoclaving at 121 °C for 20 min caused a reduction in trypsin inhibitor activity of both faba beans (50.40 and 84.03%), but combining with dehulling and/or soaking caused an increase of the trypsin inhibitor activity vs. autoclaved seeds. The increase by dehulling demonstrated that trypsin inhibitors are mainly located in the seed cotyledons. In contrast, Choi et al. [53] observed an increase in trypsin inhibitor activity levels by autoclaving at 121 °C for 15 min in the adzuki bean (*Phaseolus angularis*), chickpea (*Cicer arietinum* L.), hyacinth bean (*Lablab purpureus* L.), black-eyed pea (*Vigna unguiculata* L.), pigeon pea (*Cajanus cajan* L. Millsp.), bambara groundnut (*Vigna subterranea* L. Verdc) and soybean (*Glycine max* L.). The exception was mung bean (*Vigna radiata* L.), which showed a 35% reduction.

Autoclaving of legumes resulted in an efficient thermal process in a reduction or even total inactivation of the trypsin inhibitors (22–100%) except for some underutilized legumes that, after this thermal treatment, exhibited an increase in their inhibitor activity. It was necessary to autoclave at 121 °C for 30 min to the complete inactivation of trypsin inhibitors in beans, while it was not possible in faba beans treated under the same autoclaving conditions (22–85%). These differences could be due to the different isoforms present in each legumes that can differ even between varieties [31]. Therefore, the efficiency of this process in the reduction of trypsin inhibitor activity depends on the legume type and processing time (15–45 min)**.** The low remaining trypsin inhibitor activity exhibited after autoclaving in legumes included in this review would have a positive effect in human health (1 to 1.5 TIU/mg) [74].

### 4.5. Autoclaving Effect on the Phenolic Compounds of Pulses and Their Antioxidant Activity

The phenolic compounds of legumes were significantly affected by autoclaving with respect to raw materials. However, several authors have reported both an increment [58,78,79,80,81] and a decrease [5,47,48,55,61,64,68,69,78,82,83,84], depending on the type of legume, conditions and the detection methods used to determine their amount [5]. It is known that high temperatures provoke polymerization and/or decomposition in the structure of aromatic rings of polyphenols, which makes their quantification difficult [85,86]. Moreover, contact with water at high temperatures could increase the solubility of polyphenols, thereby increasing their release into the cooking water [87]. As more water volume was used for cooking beans, higher loses of polyphenols were observed [88].

The phenolic content in the lablab bean was reduced under pressure cooking [61,86]. Such reduction can be explained by a lixiviation phenomenon that drives phenols into cooking water and by their being bound to other compounds, forming insoluble complexes [89]. The percentages of total free phenolic reduction are extended by the duration of processing, varying from 31% for 15 min to 85% for 45 min, and from 28 to 72% for tannins [61]. Vijayakumari et al. [84] reported that autoclaving seemed to be the most efficient processing for reducing polyphenol and tannin contents of *Vigna sinensis* and *Vigna aconitifolia* seeds compared to soaking and cooking. This loss of phenolic contents by autoclaving may also be related to the interaction of polyphenols with other components of seeds, such as insoluble tannin-protein complexes [82]. Pedrosa et al. [5] observed that different effects after industrial autoclaving (116 °C, 42 min) depend on the methods (spectrophotometric or HPLC) used to determine the content of total phenols in two varieties of *P. vulgaris* (var. Almonga and var. Curriquilla). A drastic reduction (70%) in phenolic content was observed by HPLC in both tinned beans, while with spectrophotometry, tinned Almonga showed a significant increase (12%), and tinned Curruquilla a significant decrease (9%). In addition, according to the HPLC results, industrial processing induces qualitative changes among varieties and processed vs. raw beans. A lower percentage of reduction in the total phenolic content of beans (var. Raba and Warta) was observed under shorter processes (7–12 min) and without previous soaking (26% and 18%, respectively) [59]. The combination of soaking and autoclaving caused a significant reduction in tannin contents of kidney bean, and the combined effect was significantly higher than cooking or soaking alone [55]. Siah et al. [63] found light losses (31– 48%) of phenolic compounds by soaking *V. faba*. A decrease also was observed in autoclaved *Lens culinaris* seeds var. Anita and Tina (32 and 30%, respectively), and even lower in pea var. Milwa (14%) [59]. A 50% reduction in tannins was observed in autoclaved lentils and chickpeas autoclaved for 35 min [47,64], a 60% loss in autoclaved faba beans autoclaved for 30 min [48] and a 35.94% loss in lentils autoclaved for 35 min [64]. Autoclaving processes (115 °C for 20 min, including pre-soaking for 12 h) of several faba beans (*V. faba* var. Rossa, Nura and TF (Ic*As)*483/13 line) reduced total phenolic (35–63%) and flavonoid (33–42%) contents and their antioxidant activity, as evaluated by different methodologies (ORAC: oxygen radical absorbance capacity assay; TEAC: Trolox equivalent antioxidant capacity assay; and FRAP: ferric reducing antioxidant power assay). When these contents were evaluated on the autoclaved seeds separated from their respective broths, some differences were observed. These contents and their antioxidant activities in all autoclaved faba beans were lower than their respective broths. Compared to the cooking process, high pressure (autoclaving) caused more disruption to seed membranes, which allowed the release of the greater amounts of some components into the cooking broth. In addition, autoclaving faba beans caused the disappearance of the less-polar phenolic compounds. Autoclaved beans were also darker in color compared to cooked beans, suggesting the formation of new compounds as a result of Maillard reactions [63]. The phenolic content reduction by cooking could be due to either their destruction or the formation of new insoluble components with other organic compounds [90]. The leaching of phenols could be influenced by the physical properties of the seeds, such as size, coat thickness, color, shape and hardness [91]. From a health benefit point of view, it was recommended that the legume seed be consumed together with the autoclaving broth. In contrast, several researchers reported that autoclaving increases the total phenolic content and antioxidant activity in *P. vulgaris* and *G. max* [58,80] and in other legumes (*C. arietinum, Lathyrus sativum* and Brazilian beans) [79,81]. In different varieties of *P. vulgaris* L. (kidney bean, pinto bean, black bean and borlotti bean), pressure cooking (115 °C, 20 min, pre-soaking) increased the total phenolic contents, flavonoid and ortho-diphenol content and antioxidant activity [58]. In soybean, the same pressure cooking modestly increased the total phenolic content and antioxidant capacity, and substantially increased the genistein and daidzein content. Discarding the cooking water significantly decreased the content of total phenols and their antioxidant activity in beans and soybeans. This suggested that heat could cause destruction of the seed structure, enabling the release of phenols into the cooking broths, and thereby increasing the extraction efficiency of the used solvent [58]. In addition, vegetable phenolic compounds are generally bound covalently to amine functional groups, and therefore heat treatment can hydrolyze them, increasing their extractability [78]. Osman [83] also reported a significant increase in tannins in heat-treated *D. lablab* bean. Amarowicz and Pegg [24] reviewed the profile and content of phenolic compounds in different legumes and the influence of soaking, boiling, fermentation and germination on their content and their antioxidant activity. They documented both increased and decreased total phenol content in boiled or autoclaved beans, lentils or peas. The DPPH activity was decreased, while the ORAC values of boiled legumes decreased, but increased in autoclaved legumes. Yeo and Shahidi [42,92] reported the effect of boiling on total, soluble and insoluble phenolic compounds and on their antioxidant activity in four lentil varieties. The phenolic content in the boiled samples ranged from 5.50 mg/g to 6.56 mg/g; the highest reduction corresponded to the insoluble phenols, and on average, the total phenolic compounds were reduced by 13%. The antioxidant activity was measured by means of DPPH and ORAC activities. Boiling reduced both parameters from 3% to 39% and from 75 to 17%, respectively.

Autoclaved legumes can be consider a sources of phenolic compounds and tannins, particularly beans with a tannin content up to 14.37 mg/g, followed by lentils (up to 8.2 mg/g), chickpeas and faba beans (up to 6.10 and 6.51 mg/g, respectively). In the majority of cited studies, autoclaving reduced the phenolic compounds and tannin content in legumes (14–84%) with respect to raw materials, except for some types of beans when they were autoclaved under less drastic conditions (around 115 °C). However antioxidant activity was reduced in some legumes by autoclaving but, on the contrary, in other cases they increased or were not affected. These different effects may be due to different methods used to quantify them by the authors cited, other than processing conditions. Despite these reductions, the inclusion of a serving dose of autoclaved legumes (100 g) in our diet will provide a certain amount of phenolic compounds to contribute to the recommended minimum intake of 300 mg/g, and consequently, it would maintain a beneficial effect on human health related to this kind of compound [38].

## 5. Effect of Extrusion/Cooking on the Bioactive Compounds of Pulses

Forty-three papers we selected were concerned with the effect of extrusion/cooking on the bioactive compounds; of these, 13 papers examined the effect on the content of a-galactosides, 16 studied the effect on myo-inositol phosphates, 10 reported the effect on protease inhibitors (mainly trypsin inhibitors), 13 examined the effect on the phenolic compounds (mainly total phenols and tannins) and 10 papers examined their antioxidant activity.

### 5.1. Extrusion/Cooking Process

There are different processes that allow the inclusion of pulse flours in new food products with high nutritional values and health-giving attributes. One of these processing methods is extrusion/cooking, since it is a versatile technology that allows the development of different food products such as snacks, breakfast cereals, instant soups, sports foods, baby foods, meat analogues, etc. Extruded products show good nutritional, functional and sensorial properties able to satisfy the consumer’s demands for nutritious, convenient and healthy foods adapted to the modern lifestyle [93].

The extrusion parameters used, mainly temperature (<100 °C) and moisture (<10%), shear forces, screw speed (<50 rpm) and pressure generated in the extruder (up to 200 MPa), help to knead, compress, plasticize and cook the starchy and/or proteinaceous raw materials [94]. When the modified material exits from the die of the extruder, there is an instant pressure drop that produces a puffing effect; therefore, extrusion/cooking can modify both the composition and the texture of the raw materials, producing more appealing foods [19,93]. Depending on the parameters selected, the products obtained can be very different: directly expanded, indirectly expanded, textured or co-extruded, which include food products such as ready-to-eat cereals, snacks, infant formulas, textured meat-like products, etc. [95]. In general, these kinds of products are developed with corn or rice starches. However, these products are considered to be low-nutrition and high-energy-density foods that may promote obesity, cardiovascular events or metabolic syndromes [96]. The use of pulses as ingredients in the formulation of these kinds of products is an economic way to improve their nutritional quality [13]. The high protein and dietary fiber contents of pulses make it necessary to optimize the extrusion parameters for each pulse and mixture used as raw materials. Recently, Pasqualone et al. [19] reviewed the optimal processing conditions for different legumes to obtain nutritious and well-expanded legume-based foods or extruded pulse ingredients. From a nutritional point of view, extrusion/cooking has positive effects, since it causes starch gelatinization, increases the soluble dietary fiber, reduces lipid oxidation and improves the retention of nutritive components, flavors and colors of extrudates [96,97]. Extrusion/cooking also affects the content of the bioactive compounds/anti-nutritional factors present in the raw materials. In the literature, there are studies that reported either their increase or their reduction, mainly depending on the raw material and the extrusion parameters; moreover, not all the bioactive compounds were affected to the same extent under the same extrusion conditions [13,18,37,65,93,98,99,100,101,102,103,104]. As has been stated previously in this review, in many cases, the elimination of some phytochemicals is not desirable, since small amounts are able to produce beneficial health effects against different chronic diseases [3,9,21,105].

The effect of extrusion/cooking on the content of the different bioactive phytochemicals reviewed is shown in (Table 2 and Table 3).

### 5.2. Extrusion/Cooking Effect on the α-Galactosides of Pulses

Regarding the extrusion effect on the content of α-galactosides, the results found in the literature are controversial, since contradictions are reported for pulse extrudates. Moreover, the different oligosaccharides may be affected in a different way [75]. The extent of these modifications depends not only on the extrusion parameters, but also on the raw materials submitted to the processing. Varela et al. [115] studied the changes in total galactoside content in different pulse/corn starch mixtures extruded at 150 °C and 20% moisture. They reported an increase in the total galactoside content (around 13%) in extruded pea blends, but a decrease in kidney bean (3%) and faba bean (47%). Verbascose showed a reduction from 26% (lupin) to 90% (faba bean) after extrusion, while this oligosaccharide increased by 15% in extruded pea. After extrusion, the stachyose content increased from 11% to 35% (pea and faba beans, respectively), but decreased 15%, 18% and 66% in lupin, chickpea and common bean, respectively. On other hand, extruded pea, common bean and chickpea showed an increase in their raffinose content (23%, 97% and 2%, respectively), but a reduction was reported for faba bean (61%) and lupin (20%). In general, the observed increase has been related to the release of carbohydrates from the food matrix due to the cell damage that occurs during extrusion, which improved the oligosaccharide extraction. A similar trend was reported by Alonso et al. [106] in comparison to the raw seeds, in which the content of raffinose, stachyose and verbascose were reduced in extruded kidney bean at 150 °C and 20% moisture; however, in extruded peas (under the same conditions) only stachyose showed a drop (20%) in its content, and raffinose and verbascose increased their content (4% and 34%, respectively). These authors reported that some sugars can interact with proteins (Maillard reaction), resulting in reduced extractability of these sugars, which may explain the reduction observed after extrusion; whereas hydrolysis reactions that can occur during extrusion can lead to an increased content of some sugars [99]. Berrios and Pan [121] reported a reduction of the α-galactosides in extruded black bean, although the extent of this reduction was different according to the extrusion conditions used; however, Berrios et al. [116] reported that the extrusion of black bean with different percentages of sodium bicarbonate (160 °C, 20% moisture) did not significantly modify the content of α-galactosides. Berrios et al. [111] found both an increase and a decrease of raffinose and stachyose in extruded peas, chickpeas and lentils at 170 °C and 17% moisture. In extruded peas, raffinose and stachyose showed a reduction (52% and 75%, respectively) compared to the raw seeds; the opposite trend was reported in extruded chickpeas, where the raffinose and stachyose content increased after extrusion (25% and 33%, respectively). Ai et al. [65] studied the extrusion effect at 120 °C and 25% moisture, and 140 °C and 30% moisture, on four bean varieties. All the bean varieties showed the same trend, with extrudates containing lower amounts of raffinose but higher amounts of stachyose than the raw samples. Borejszo and Khan [107] reported a reduction of raffinose and stachyose from 44% to 60% in extruded pinto bean, with a higher reduction at a higher extrusion temperature (110–163 °C). Extrusion of pre-soaked pinto bean seeds at a low temperature (85 °C) and 36% moisture [108] resulted in a 20% decrease in raffinose and a 10% decrease in stachyose content compared to the raw seeds. The different reduction trends observed may be due to differences in raffinose and stachyose solubility. Some authors [18,97,100,103,120] have developed pulse-based snacks fortified with different fiber sources with good sensorial acceptability. Morales et al. [120] reported that the snacks formulated with lentils presented a higher α-galactoside amount than those of the unprocessed mixes, from 2% to 31% depending on the formula ingredients (wheat bran or corn and apple fibers). This effect could be due to a more effective extraction of sugars in the extruded food matrix. Verbascose decreased in almost all formulations (from 1% to 21%), while stachyose and raffinose increased from 4% to 45% and from 8% to 55%, respectively. This could be due to the partial hydrolysis of the longer oligosaccharide under the high temperature (160 °C) and pressure of the extrusion process, which produced fewer raffinose family oligosaccharides. Ciudad-Mulero et al. [97] also developed lentil-based snacks, but enriched with different percentages of a nutritional yeast (*Saccharomyces cerevisiae*) extract using two extrusion temperatures (140 °C and 160 °C) at 17% moisture. After extrusion, with the exception of the sample formulated with 12% yeast, the samples showed a significant increase in total α-galactosides, with this increase being higher in the samples extruded at 160 °C. While raffinose and verbascose increased in all the samples, the stachyose content did not show a consistent tendency. Arribas et al. [18,100] developed snacks formulated with different percentages of rice, and pea or bean, fortified with whole carob bean flour and extruded at 125 °C and 20% moisture. All the extrudates showed higher content of α-galactosides than the non-extruded samples. Rice/bean and pea/rice snacks contain up to twice as many α-galactosides than their corresponding controls, and the highest pulse content produced the highest sugar increase. The sugar pattern was verbascose > stachyose > raffinose in the rice/pea extrudates and stachyose > raffinose in the rice/bean extrudates. In the pea-based snacks, on average, extrusion produced an increase of verbascose and stachyose of around 52% and around 100% of raffinose; whereas in bean-based snacks, the increase was around 48% and 200% for stachyose and raffinose, respectively. The authors related these increases to the higher extractability of sugars from the extruded matrix and to a hydrolysis of the sugars during the extrusion. The data evaluated show that the content in α-galactosides in the extruded products from different legume seeds ranged from 15.2 mg/g to 47.8 mg/g in beans, from 14.38 mg/g to 44.21 mg/g in chickpeas and from 12.82 mg/g to 14.02 mg/g in lentils. Only one paper studied the a-galactosides content in extruded faba bean and lupin seeds, with their content after extrusion/cooking being 31.66 mg/g and 83.76 mg/g, respectively. Despite many authors reporting an influence of the seed variety on the results, only five papers detailed the variety used in their studies. We can conclude that, in general, the α-galactosides are reduced after extrusion cooking up to 68%, depending on the legume seed and the extrusion conditions. In different types of common beans, the reduction reported in eight papers was between 3% and 33%, but five papers reported an increase in the α-galactosides content, from 2% to 97%. The reduction documented for chickpeas was from 17% to 68%; regarding the extrusion effect on lentils, both a decrease from 60% to 63% and an increase from 2% to 31% has been reported. In extruded peas, the a-galactosides content was reduced from 1.2% to 15%, although two papers reported an increase from 13% to 35%. Faba bean and lupin were reduced by 47% and 23%, respectively. Many of the extruded legumes are of great interest in the development of snacks or breakfast cereals; therefore, considering one serving of these types of extruded product (40 g) can supply 483–560 mg of α-galactosides for lentils, 938–1876 mg for peas, 5575–1768 mg for chickpeas and 608–1912 mg for beans. All the extruded legumes are a good and adequate source of galactosides able to exert a healthy influence [25].

### 5.3. Extrusion/Cooking Effect on the Myo-Inositol Phosphates of Pulses

The inositol phosphates are also affected by extrusion. A thermal hydrolysis of the phytic acid (IP6) into less phosphorylated forms has been reported, with the phytic acid reduction being higher as the extrusion temperature is increased [27,30,114]. As phytate can bind other macromolecules, the formation of insoluble complexes may occur during extrusion, which reduces their extractability, thus explaining its reduction [122]. This reduction could improve the mineral bioavailability and allow the health roles associated with the inositol phosphates. Most of the literature reported a decrease in the total inositol phosphates and the phytic acid (IP6), although the extent of this reduction varied with the raw materials and the extrusion conditions. El-Hady and Habiba [104] observed a reduction in IP6 content (2–9%) in extruded faba bean, pea, chickpea and kidney bean, with the higher temperature (180 °C) and moisture (22%) used being more effective in decreasing phytic content than the lower ones (140 °C and 18% moisture). A similar trend was reported by Anton et al. [37] in an extruded navy or small red bean/corn starch blend. They observed an overall reduction in total inositol phosphates of 44%. A higher reduction of phytic acid has been reported by Rathod and Annapure [114]. These authors found that the increase in extrusion temperature (140 °C, 160 °C and 180 °C) and moisture (14%, 18% and 22%) produced a decrease in phytic acid up to 99%. In contrast, Lombardi-Boccia et al. [123] observed that the legume phytate content in mottle and white beans, faba beans, chickpeas and lentils was not affected by extrusion at 130 °C and 28% moisture. In extruded chickpeas at 130 °C and 14.5% moisture, Poltronieri et al. [112] observed a reduction in neither total inositol phosphates nor phytic acid; however, they observed a significant reduction in IP5. Alonso et al. [99] also reported that the phytate content did not change significantly in extruded breakfast meals formulated with 30% bean flour and broken rice [14], or in extruded peas at 145 °C and 25% moisture. Morales et al. [119] reported a general reduction in total inositol phosphates from 14% to 50% in snacks produced at 160 °C and based on lentils and different fibers, depending on the formulation. The addition of different types of fibers produced different matrixes with a visco-rheological behavior during extrusion; thus, the extrudate formulas containing corn and apple fiber showed a non-significant reduction. IP6 showed the highest reductions, while IP5 and IP4 forms increased their content, on average, by 13% and 26%, respectively. Similarly, Alonso et al. [106] reported an increase in IP5 in extruded kidney beans (5.2-fold) and peas (1.5-fold) compared to raw seeds. However, Ciudad-Mulero et al. [97] reported not only an IP6 decrease ranging from 35% to 48% for lentil/yeast samples extruded at 140 °C and 160 °C, but also an IP5 and IP4 reduction from 19% to 51% and 17% to 43%, respectively. Hegazy et al. [113] produced snacks based on germinated and dehulled chickpeas (up to 30%) and corn. The extrusion at 180 °C and 16% moisture decreased the phytic acid content by 41–46% compared to extruded corn as a control. After extrusion at 150 °C and 20% moisture of different pulse/corn starch blends (80/20), Varela et al. [115] observed both an increase and a reduction of the total inositol phosphate content, depending on the pulse type. While extruded bean, chickpea and faba bean showed a significant reduction (7%, 8% and 36%, respectively), pea and lupin showed a significant increase in the total inositol phosphate content (3% and 24%, respectively). Arribas et al. [18,100] documented a reduction of phytic acid by 10% in extruded snacks (125 °C, 20% moisture) formulated with different proportions of rice/bean or pea/carob fruit; moreover, an increase of 16–70% of the less phosphorylated forms (IP4–IP5) was observed. These authors also reported that the higher legume content caused the higher phytate reduction. Thus, from a nutritional and health point of view, extrusion would be a good processing method to reduce phytic acid content and obtain the benefits of the less phosphorylated forms. Yağci et al. [118] reported the effect of extrusion and locus bean gum content on the phytic acid content of fermented chickpea-yogurt blends. Extrusion was developed at 140 °C and 150 °C with 17% moisture, and the gum content varied from 2.5% to 4%. The phytic content in the fermented blends was reduced by 31.3–41.3% after the extrusion treatment. The highest reduction was obtained in the blends containing 4% of locus bean gum extruded at 150 °C, and the lowest one corresponded to the blends with 1% of gum extruded at 150 °C.

In addition, by comparing the results reported in the literature reviewed, the content of myo-inositol phosphates ranged from 0.54 mg/g to 16.59 mg/g in beans, from 1.00 mg/g to 12.6 mg/g in chickpeas, from 6.05 mg/g to 6.86 mg/g in faba beans, from 0.08 mg/g to 0.57 mg/g in lentils and from 3.14 mg/g to 11.23 mg/g in peas. Regarding the effect of extrusion/cooking on the myo-inositol phosphates content, in general a reduction of its content was observed, with a high variability in the reduced percent (from 1% to 99%), although in five reviewed papers, an increase was reported. The inositol phosphates content decreased from 1% to 21% in beans, from 1% to 53% in chickpeas, from 95% to 99% in lentils and from 6% to 21% in peas; however, an increase was documented in the myo-inositol phosphates content of 3% for chickpeas, lupin and peas, and of 22% for beans. Assuming a protein content for these extrudates of around 20%, one serving (40 g) of the extruded products, similar to a snack than can supply less than 10 mg/g to improve the mineral absorption [30], were the extruded lentils (0.4–2.85 mg/g protein), some of the extruded chickpeas (5 mg/g protein), beans (0.27 mg/g protein) and faba bean (5.99 mg/g protein).

### 5.4. Extrusion/Cooking Effect on the Protease Inhibitors of Pulses

Protease inhibitors are proteinaceous heat-labile compounds, so their content is significantly reduced by extrusion cooking; depending on the seed, the formulation of the raw flours and the extrusion parameters used, the extent of the reduction will vary, even to zero [122]. Extrusion at 150 °C and 20% moisture of some pulse/starch mixtures produced a different percentage of reduction in the trypsin inhibitory activity. The lowest reduction corresponded to lupin blends (77%), followed by chickpea (89%), faba bean (93%), pea (95%) and bean (99%). These differences could be related to the presence of different inhibitor isoforms, some of which are more resistant to thermal treatment. This fact was confirmed by zymogram gels. Other authors [18,37,100,104] reported that the trypsin inhibitors were abolished in corn starch-based snacks with navy or red bean produced at 160 °C and 22% moisture, as well as in pre-soaked and extruded (140 °C–180 °C, 18–22% moisture) pea, chickpea, faba bean and kidney bean, and in extrudates (125 °C, 20% moisture) based on rice, pea or bean and carob bean mixtures. Zamora [110] reported that the extrusion of *Canavalia ensiformis* (155 °C, 20% moisture) affects the chymotrypsin and trypsin inhibitor activity by 99% and 95%, respectively. In contrast, other authors [99,124] reported that chymotrypsin inhibitor activity was lowered less extensively than trypsin inhibitor activity in pea cv. Ballet when extruded at 145 °C, 25% moisture (65% vs. 95%), and in faba bean var. Aguadulce when extruded at 140 °C and 40% moisture (42% vs. 92%). Rathod and Annapure [114] reported a drastic reduction in trypsin inhibitors (up to 99.5%) in extruded split lentils; as the extrusion temperature (from 140 °C to 180 °C) and moisture (from 14% to 22%) increased, a higher increase in the removal of trypsin inhibitors was observed. Reductions in the trypsin inhibitor activity from 93% to 95% have been reported in extruded lentil enriched with nutritional yeast at 140 °C and 160 °C. The highest reduction corresponded to the samples with the highest lentil content, while the higher yeast inclusion produced the lower trypsin inhibitor activity reduction [103]. Morales et al. [120] observed different trypsin inhibitor content in extruded lentil-based blends enriched with different types of dietary fiber. After extrusion (160 °C, 17% moisture), all samples presented a reduction in the trypsin inhibitor content, although the highest decrease (97%) corresponded to extruded lentils plus apple fiber and/or corn flour, and the lowest one (93%) corresponded to blends of lentils with wheat bran. Extrusion cooking of pigeon peas and African yam beans at 100 °C and 140 °C and 16% moisture significantly reduced the protease inhibitor activity (up to 98%); however, the trypsin activity of bambara groundnut extruded at 100 °C was not modified [125]. The extrusion of fermented chickpea-yogurt and locus bean gum blends reduced the trypsin inhibitor activity in the range of 77–79%, with the higher reduction corresponding to the extrudates obtained at 150 ºC with a 4% of gum content [118].

The protease inhibitors are thermo-labile compounds; therefore, they were reduced from 77% in lupin to 99% in beans. These differences are related to the different thermo-stability of the different isoforms present in each legume [31]. However, in general, protease inhibitors were not detected after extrusion. The higher activity was determined in chickpea (2.52 TIU/mg), and the lowest one in lentil (0.013 TIU/mg). Bean, faba bean, pea and lupin showed protease activity of around 0.25 TIU/mg. One serving of 40 g can supply 0.52–9.6 TIU, and since these amounts are below the concentration to impair protein digestibility, they are considered as safe products.

### 5.5. Extrusion/Cooking Effect on the Phenolic Compounds of Pulses and Their Antioxidant Activity

Considering the effects of extrusion on the content of phenols, both increases and decreases in their content have been reported, mainly depending on the type of phenolic compounds studied. In general, decreases have been related to decarboxylation of phenolic acids and/or their polymerization during extrusion, which reduce their extractability; whereas increases have been related to the phenols released from the cell-wall matrix; consequently, the antioxidant activity associated with the phenols is also affected by the extrusion. Korus et al. [109,126] studied the effect that extrusion parameters (two temperatures and two moistures) had on the phenols and their antioxidant activity in five cultivars of dry common bean. They observed that extrusion at the lower temperature (120 °C) and the higher moisture (20%) retained the highest proportion of phenols. In comparison to the raw samples, a reduction in the total phenols and the antioxidant activity was observed, and these changes were cultivar-dependent. On average for the two temperatures used, the Rawela cv. (dark-red bean) increased its total phenol content (14%) after extrusion, while Toffi (black-brown bean) and Tip-top (cream bean) varieties decreased their content (by 19% and 21%, respectively). The different phenolic-compound contents after extrusion also are affected by the cultivar. Other authors [102,104,114] have also reported a significant decrease in the phenolic-compound content and the antioxidant activity of whole peas, faba beans, chickpeas and kidney beans, and the extent of this reduction was linked to the extrusion parameters used. Changes in the phenolic composition of extrudates, based on cereal/pulses or vegetable/pulse blends, have also been studied, since the food matrix highly conditions phenol bioaccessibility. Delgado-Lincon et al. [117] examined the effect of five extrusion temperatures (150 °C–190 °C) and four moisture levels (14.5–18%) on the phenol content and the antioxidant activity of a bean/corn blend (60/40). They observed a significant decrease in total phenols, flavonoids and antioxidant capacity, and they concluded that extrusion at 142 °C and 16.3% moisture retained the highest amount of total phenols and flavonoids, and showed the highest antioxidant capacity. Anton et al. [37] reported a significant decrease in the total phenol content and antioxidant activity of corn-starch snacks containing two different bean flours. The red bean/corn snack showed a higher reduction in total phenol content and antioxidant activity (around 70% and 65%, respectively) than the navy bean/corn snacks (around 10% and 22%, respectively). Lentil and nutritional yeast extruded at 140 °C and 160 °C showed decreases in total phenolic compounds, individual phenolic compounds and antioxidant activity compared to the raw blends. The observed decrease was correlated to the extrusion temperature. Also, a reduction in the tannin content was reported by Carvalho et al. [14] in extruded rice/bean flour blends, by Alonso et al. [91,104] in pea and kidney bean meals and by Rathod and Annapure [114] in split lentils, reaching a reduction of up to 50%, 92% and 99%, respectively. Conversely, other authors reported increases in both total phenol and antioxidant activity; for example, in extruded lentil flours enriched in fibers, Morales et al. [119] found an increase in total phenolics, as well as in most polyphenolic fractions (except in flavonols), with an increase in antioxidant activity. Arribas et al. [100] reported an increase in tartaric esters (on average 11%), anthocyanins (on average 24%) and total phenol content (on average 36%), while flavonoids did not vary significantly in different extruded rice/bean/carob flour formulations compared to their raw counterparts. A slight increase in the antioxidant activity (on average 5.4%) was observed, and the antioxidant activity of the extrudates was positively correlated with their phenolic content. Díaz-Batalla et al. [127] reported that extrusion at 150 °C of *Prosopis laevigata* (legume tree) seeds reduced the content of total phenolic compounds by 3%, but increased the DPPH radical scavenging capacity by 2.30%. They did not observe an increase the content of Maillard reaction product compared to the raw samples. Hegazy et al. [113] reported an increase in the total phenolic content (1.92–7.94%) and antioxidant activity (1.07–5.55%) in corn snacks enriched with different proportions of germinated chickpea and tomato pomace after extrusion. The selection of the raw materials, their blends with other food ingredients and the selection of the extrusion parameters (mainly temperature and moisture) are very important in order to obtain end-products containing adequate amounts of all types of phytochemicals with health-giving benefits.

In summary, the phenolic compound and tannin content was mainly reduced after extrusion by up to 99%, although a high variability can be observed depending on the seed and the extrusion conditions, as well as the method of analysis used. The antioxidant activity was only determined in nine papers; however, it is difficult to compare the results due to the different methods used (DPPH, ORAC, TBARS, etc.). The phenolic-compound content in extruded beans was reduced up to 74% and 70% for total phenols and tannins, respectively; however, one paper reported an increase in the total phenol content in extruded beans from 30% to 38%. Four papers reported an increase in the antioxidant activity, and five papers described its reduction. These differences are mainly related to the different methods of analysis. One serving (40 g) of the extruded product can supply more than the minimum daily dose (300 mg) of total phenolic compounds to obtain health benefits.

## 6. Effect of Cold Extrusion on the Bioactive Compounds of Pulses

Thirty-seven papers were reviewed in this section. From them, 16 studied the elaboration of pasta by cold extrusion, and the effect of the process and/or its subsequent cooking necessary for its consumption. Two papers evaluated the effect of the cooking process on galactosides, while three of them studied the modification in the inositol phosphate content, the inhibitor protease activity and/or the phenols content after the cooking process. Furthermore, four papers showed the effect of the cooking process on the antioxidant capacity.

### 6.1. Cold Extrusion Process

Cold extrusion is the standard process used for pasta production. It is a process similar to extrusion/cooking, with a single screw and high pressures, below 50 °C (in comparison to the temperatures in extrusion/cooking (Section 5), which are above 100 °C) [128]. In this type of extrusion, the product is made without cooking, so that the raw materials are subjected to minimal modifications both by friction and temperature [129].

The conditions used in extrusion/cooking might not be ideal, since they reduce the content of thermolabile compounds such as vitamins, functional proteins and flavors that can be incorporated into the formulation. However, cold extrusion allows these to be kept in the final product [130]. Pasta consumption is very high due to its palatability, long shelf life and simplicity of cooking, in addition to its nutritional characteristics [131]. The process of making pasta consists of transforming the flours into pasta in a homogeneous way; to achieve the required texture, the flour is hydrated by mixing, and then it is extruded. There are four different stages in the preparation of pasta. First, the kneading, which consists of adding water to the mixture of flours used for the preparation of the pasta. The final dough obtained contains an average moisture between 30% and 32% [132,133]. At this stage, the mixing is carried out for approximately 10 min, avoiding the incorporation of air into the mass. The presence of small air bubbles can weaken the structure of the pasta and activate enzymes [134]. Second, the cold extrusion process, during which the moisture content of the dough is approximately 28%. The extrusion process occurs at a low temperature and with continuous pressure along the worm screw, ensuring that a temperature of 50 °C is not reached to avoid any deterioration of the proteins (as this would reduce the quality of the pasta during subsequent cooking). The dough comes out in a tube (the exit hole through which the dough comes out has the desired shape of the pasta: spaghetti, fettuccine, macaroni, etc.), where a minimum expansion takes place. The third step is drying, with the aim of producing a resistant and stable pasta. The moisture on the surface of the pasta is reduced by hot air currents, creating a moisture gradient within the pasta [135]. The drying cycles, during which the pasta acquires its final color, are finished by reaching a moisture content of less than 12% [136]. Cold extrusion is an ideal process to produce pasta fortified with legumes, although this fortification is a new process. The principal studies in the literature showed the fortification of wheat flour with legumes such as soya, pea, lentil, chickpea, lupin and bean [137,138,139,140,141,142,143,144,145,146,147,148,149,150,151,152,153,154]. These authors revealed the modifications observed in the pastas fortified in comparison to the control pasta (in general, durum wheat semolina). However, only some studies showed the differences between the bioactive compounds in the uncooked and cooked fortified pasta.

The effect of cold extrusion on the content of the different bioactive phytochemicals reviewed is shown in (Table 4 and Table 5).

### 6.2. Cold Extrusion Effect on the α-Galactosides of Pasta Fortified with Legumes

Torres et al. [146] produced pasta products based on whole durum wheat semolina fortified with proportions of 5%, 10% and 12% of fermented pigeon pea (*C. cajan*). Fermentation brought about an 82% reduction in α-galactosides in the seeds used, since the oligosaccharides were hydrolyzed by α–galactosidase and invertase either as an enzyme or from microorganisms present. Taking into account the previous fact, this study showed that the pasta fortified with 10% fermented pigeon flour did not report α-galactoside content (raffinose, stachyose and verbascose), as with the control pasta analyzed. Nevertheless, Laleg et al. [155] showed that the content of total α-galactosides ranged from 44 to 57 mg/g (d.m.) in the uncooked pasta, higher in the 100% lentil pasta than the 100% faba pasta and the 100% black-gram (*Vigna mungo*) pasta. The stachyose and verbascose were present in the oligosaccharide profiles in the uncooked pasta, and raffinose was the minor oligosaccharide in the pasta studied. On the other hand, after the cooking process, the α-galactoside content was reduced from 41–73% because of the heat hydrolysis or leaching in the cooking water. The optimal cooking time was a parameter that determined the concentration of soluble compounds such as α-galactosides in the pasta ready to eat. This effect was observed in the cooking of fettuccine based on rice and fortified with two different legumes (bean (*P. vulgaris* L.) and carob fruit (*Ceratonia siliqua* L.)) [148]. The cooked pasta showed around 4–27 mg/g content of α-galactosides, while the uncooked pasta showed around 10–57 mg/g. The difference observed (about 40–70%) could be related to the water solubility in the cooking process. These compounds migrated to the cooking water and were discarded. Taking into account the reviewed data, we can conclude that the cooking process after the preparation of the pasta by cold extrusion modified the content of α-galactosides, reducing it between 21 and 82%, in pasta elaborated with bean/carob fruit and 100% legume, respectively. The content of α-galactosides ranged in these samples between 4 and 57 mg/g. In addition, it is important to note that the processing reduced but did not eliminate these compounds, so their consumption would be beneficial to human health. The consumption of one serving of cooked pasta (70 g) will provide around 280–2380 mg of α-galactosides. This range allows the consumption of a quantity close to the one that has been observed to convey benefits on the intestinal flora [25].

### 6.3. Cold Extrusion Effects on the Myo-Inositol Phosphate Content of Pasta Fortified with Legumes

Torres et al. [153] reported that pasta products fortified with pigeon pea had a higher content of phytic acid than the control produced with 100% semolina. On the other hand, in the pasta fortified with germinated *C. cajan* in proportions of 5%, 8% and 10%, the phytic acid presented twice the amount than the control pasta [149]. Herken et al. [154] analyzed the phytic acid content using the colorimetric method in unprocessed, germinated, fermented and cooked pasta made from cowpeas. These authors showed that the germination, fermentation and thermal processes reduced the phytic acid content from 13.3% to 15.2%, with respect to the unprocessed samples. The 100% legume pasta revealed phytic acid contents from 14.5 to 18 mg/g in the uncooked pasta. A slight reduction in this content was observed in the cooked pasta (from 12.8 to 15 mg/g) related to the corresponding flours. A low content in phytic acid in the cooked pasta was associated with high iron bioavailability and a general nutritional quality [155]. On the other hand, Arribas et al. [157] showed a slight increase (*p* > 0.05) in the total inositol phosphate (the different IP forms included phytic acid or IP6), which can be explained by losses during cooking; these compounds migrated to the cooking solution, which caused changes in the percentage of the specific components on a dry-matter basis. Bilgicli et al. [138] studied the phytic acid content in pasta based on wheat, maize and rice flour fortified with soya and chickpea grains. They showed ranges of phytic acid from 7 to 9 times higher than the control noodle made from wheat flour.

In this case, the reduction in inositol phosphate content, regardless of the seed used in the production of the pasta, was around 15%. In the cooked pasta reviewed, the phytic acid content ranged from 3 to 574 mg IP6/g in the rice pasta fortified with bean and carob fruit and in germinated and fermented cowpea flours, respectively. Therefore, the consumption of one serving (70 g) of pasta would be associated with the health benefits of these compounds [3,9,22,23].

### 6.4. Cold Extrusion Effects on the Protease Inhibitors of Pasta Fortified with Legumes

Several authors have studied the inhibitor protease content in pasta fortified with different proportions of legumes. Torres et al. [146,149] showed, in pasta based on semolina and fortified with fermented and germinated *C. cajan* seed in proportions of 5% or 8% and 10% or 12%, respectively, that the trypsin inhibitor activity in the fermentation of pigeon pea seeds affected trypsin inhibitor activity and caused a reduction of 39%. This was not detected in the pasta produced with 8–10% fermented *C. cajan.* However, in the pasta with 10% germinated *C. cajan*, the trypsin inhibitor activity determined was 1.57 TIU/mg, which is an insignificant amount when compared to the trypsin inhibitor activity value found in the germinated flour (15.9 TIU/mg). The difference between the two processes, fermented and germinated, revealed that the fermentation of pigeon pea produces a significant reduction in the trypsin inhibitor activity, and the inclusion of this flour in small quantities produced pasta without trypsin inhibitor activity. That said, however, the use of germinated pigeon pea flours could be associated with the positive effect of these bioactive compounds.

Frias et al. [151] produced macaroni pea (*P. sativum* L.) products, and the trypsin inhibitor activity analyzed revealed that as the cooking time of the pasta increased, the reduction of this activity was 48–59%. Taking this into account, the cooked pasta had lower reported contents than the uncooked pasta, since heat inactivated the trypsin inhibitor activity, although it increased the legume protein digestibility. This effect was observed in pasta made with 100% legume [147]. The analyzed samples ranged from 7.8 to 11.3 mg/g, but the cooked pasta revealed five times lower trypsin inhibitor activity, and the cooking process in legume pasta was reduced by 38% to 96% of this activity. Thermal processing of legumes can be applied to totally or partially reduce the protease inhibitor activities because they are thermo-labile compounds. However, Arribas et al. [148] showed, in the fettuccine made with rice, bean and carob, a slight increase (*p* > 0.05) in trypsin and chymotrypsin inhibitor activities after the cooking process, in comparison to the uncooked samples. This could possibly be due to the short cooking time applied (on average 3 min) and to the different heat-sensitivity of both types of protease inhibitors.

The cooking process of the pasta showed in general a reduction in the protease inhibitor content (around 50–80%). However, in the pasta formulated with bean and carob fruit included in the review, a small increase in this content was observed. The protease inhibitor activity after the thermal process ranged between 2–17 TIU/mg and 3–17 CIU/mg. The cooking process (100 °C, 3–10 min) was not so severe to eliminate these compounds, unlike in other products. One serving (70 g) of the pasta would supply from 140 to 1190 TIUs and from 210 to 1190 CIU. These amounts are below the content reported to impair protein digestibility (2000 CIU per day), and most of the reviewed pasta contained amounts in the range of 25–800 CIU/day, which is able to exert the health benefits associated with these bioactive compounds (Section 3.3).

### 6.5. Cold Extrusion Effects on the Phenols and Their Antioxidant Activity of Pasta Fortified with legumes

Phenols and their associated antioxidant activity are some of the most commonly analyzed features of products made by cold extrusion. Pasta fortified with 1, 2, 3, 4 and 5% carob fruit (*C. siliqua* L.) revealed higher contents of phenols using the Folin–Ciocalteau reagent (3.21–4.8 mg gallic acid equivalent (GAE)/g d.m.) and antioxidant activity analyzed by chelating power (CHEL), ABTS radical scavenging assay, ability to inhibit lipoxygenase and ferric-reducing antioxidant power (FRAP), more than the control sample made with common wheat flour [163]. Seczyk et al. [164] studied the fortification from 1%–5% of carob pod in pasta based on durum wheat semolina. The results obtained demonstrated that the carob pod flour increased the phenolic content determined (5.3–12.2 mg GAE/g d.m.) and the antioxidant activity analyzed (0.25–1.35 mg Trolox equivalent/g in the ABTS assay and 2.05–6.9 mg Trolox equivalent/g in the FRAP assay) showed the highest values and activity in the formulation with 5% of carob, such as in previous studies [163]. In comparison to the control, the pasta fortified with 5% carob pods revealed double the amount of phenolic content, and a three- to 18-fold increase in antioxidant activity. In the gluten-free pasta made with rice and fortified with different proportions of legumes (carob fruit 10%), the results obtained showed a total phenol content in the uncooked samples of around 2.9–7.3 mg catechin equivalent (CE)/g d.m., but after the cooking process, the total phenol content was reduced to around 17–48% (2.3–5.8 mg CE/g dw). The pasta fortified with 10% carob fruit presented a 2.5–3.2-fold higher amount of total phenols than the commercial sample made with rice and used as a control. The antioxidant activity by ORAC assay in the uncooked fettuccine revealed 5.2 to 13.7 µmol Trolox/g d.m. After the cooking process, the ORAC assay exposed 5.1–10.3 µmol Trolox/g d.m. in the fortified fettuccine. The cooking process showed a slight and non-significant reduction in the antioxidant activity [157]. These studies showed the potentialities of this legume, carob, to fortify products made with cold extrusion, since it is a good source of polyphenols and therefore of antioxidant activity, improving the health characteristics of traditional pasta based on wheat. In Turco et al. [161], pasta made of 100% pea flour, 100% red lentil flour, and 60% grass pea and 40% chickpea flour revealed that total polyphenols and flavonoids determined by the Folin–Ciocalteu method and antioxidant properties determined by ORAC were higher in the legume pasta than in the control made with durum wheat semolina. The pasta made with 60% grass pea and 40% chickpea was the formulation with the highest total polyphenol content, followed by red lentil pasta and pea pasta. All the experimental formulations revealed higher content than the pasta made with durum wheat semolina (control). Turco et al. [159], in pasta based on semolina and fortified with 35% faba bean, showed a total phenol content around two times higher compared to the control. Also, the higher content of flavonoids and antioxidant activity found in the ORAC assay was lower in the control pasta. Torres et al. [153,160] showed that the tannin content in the pasta fortified with 10% germinated pigeon pea flour (0.19 g galic acid/100 g d.m.) and fortified with 10% fermented pigeon pea flour (0.28 g galic acid/100 g d.m.) was lower than the control elaborated with 100% semolina. The fermentation process could improve the phenolic content in relation to germination. The antioxidant activity was analyzed in the fermented samples, and the results revealed that the fortification increased the total antioxidant activity by around 80% with respect to the control sample. Herken et al. [154] showed that in samples fortified with different proportions of cowpea flour (10%, 15% and 20%), the inclusion of 20% legume, fermented or germinated, increased the total antioxidant activity (31.16–34.51µmol Trolox equivalent/g, respectively) in comparison to the control made with 100% semolina. However, it was observed that the antioxidant activity detected in the macaroni with 20% fermented flour showed lower activity than the macaroni with unprocessed flour (*p* < 0.05), and lower than the germinated ones, in contrast to other authors [153,160]. However, cooking had a negative effect on the antioxidant activity determined.

The inclusion of bean in pasta has been studied by different authors; Gallegos-Infante et al. [162] reported that the highest phenolic compound content detected was in the samples fortified with common bean flour (15–30%). Arribas et al. [157] showed the same tendency in the samples based on rice and fortified with two different legumes. The inclusion of bean in these samples increased the total phenolic content, and the antioxidant activity detected in the uncooked samples was 5.3–13.7 µmol Trolox/g, with higher legume content revealing higher antioxidant activity. After the thermal process, the same tendency was observed; however, the antioxidant activity was reduced in all formulations. Segura-Campos [146] showed in pasta made with durum wheat semolina and hard-to-cook bean protein hydrolysate (0%, 5%, and 10%) that the antioxidant activity determined by the ABTS decolorization assay increased antioxidant values. The highest antioxidant value determined was in the pasta fortified with 10% of hard-to-cook bean protein hydrolysate (31.4 mM/mg); however, the cooking process revealed lower antioxidant values (around 6.6–7.9 mM/mg sample in the pasta fortified with 5% and 10% hard-to-cook bean protein hydrolysate, respectively). In all these cases, we can conclude that the thermal process can degrade the bioactive compounds (phenols, peptides, etc.) and decrease the antioxidant activity analyzed. Spaghettis fortified with 5–10% legume hydrolysates showed that the pasta made with cowpea (*V. unguiculata*) hydrolysate revealed higher antioxidant activity (294 µmol Trolox/g), determined by the ABTS assay, than the lima bean (*P. lunatus*) hydrolysate (245 µmol Trolox/g) and the control analyzed. In general, a higher amount of legumes in the formulation increased the antioxidant activity, so the inclusion of 10% hydrolysate of *P. lunatus* in the pasta showed a twofold increase in antioxidant activity than the control, which could be due to the presence of low molecular weight peptides in the hydrolysate [158]. In commercial samples manufactured with lentils, beans and peas, these types of pasta revealed higher total phenolic compounds than cereal-based pastas, and the black lentil pasta revealed the highest total phenol content (1743.3 mg GAE/100 g d.m.). Black bean pasta, pea pasta and red lentil pasta also presented higher total phenol content than the cereal and pseudo-cereal-based pasta studied (781, 657.1 and 631.6 mg GAE/100 g d.m., respectively). After cooking, the free polyphenol fraction decreased by around 50% in all the legume pastas (323.3 to 813.5 mg GAE/100 g d.m.) in comparison to the original content in the samples, due to the solubilization in the cooking water and the thermal sensibility of these compounds [156]. Di Stefano et al. [131], in pasta based on semolina and fortified with 40% of lentil, showed around a 28% reduction of the total phenolic content after the cooking process.

The content of the total phenolic compounds in the cooked pasta was 0.93–1.42 mg GAE/g. All the data analyzed in this section showed that the cooking process reduced the content of phenolic compounds from 20–51%. The range obtained in the papers included in this review was between 0.93 and 814 mg GAE/g. in pasta fortified with 40% lentil and commercial pasta elaborated with legumes, respectively. One serving (70 g) of almost cooked pasta fortified with the legumes studied would provide an average of 0.56 g GAE/g, which is enough to obtain a healthy intake of phenolic compounds [45].

## 7. Concluding Remarks

It is well known that pulses are an inexpensive and sustainable source of nutrients, and they represent functional foods that are rich in different bioactive compounds, providing many health benefits when consumed regularly in a balanced diet. Pulses are processed prior to their consumption; therefore, even though a pulse seed contains a high amount of bioactive compounds, it does not mean that the processed pulse has a high content of the bioactive components, because their processing modifies their content. The extent of these modifications depends on the seed, the variety, the food matrix, the formulations and the technological processing. Galactosides were reduced up to 77%, 68% and 82% in autoclaved, extrusion/cooked and cold-extruded cooking samples, respectively, although some papers reported an increase in some of the extrusion/cooking processed samples. Myo-inositol phosphates were more affected by autoclaving and extrusion/cooking, reaching reduction of 2–100% and 1–99%, respectively. Cooked pasta reduced their content up to 15%. All the processes reviewed drastically reduced the protease inhibitor content (80–100%); therefore, we concluded that these samples would show an improved protein digestibility. The majority of the revised papers reported a reduction in phenolic compounds of up to 80%, 99% and 51% for autoclaved, extrusion/cooking and cold-extruded/cooked products. One serving of autoclaved legumes, extruded/cooked and cold-extruded/cooked products can supply on average 3.2, 1.8 and 1.3 g of galactosides, respectively; even though there is no recommended daily intake, according to the literature, it would be enough to obtain prebiotic benefits on the gut. Considering one serving of each product and depending on the legume seed and the processing conditions, some of the processed legumes could supply less than 10 mg/g protein, and thus improve mineral absorption. The low remaining trypsin inhibitor activity (<1 to 1.5 TIU/mg) in the processed legumes included in this review would have a positive effect on human health. One serving of a majority of the reviewed products can supply more than the minimum daily dose (300 mg) of total phenolic compounds to obtain health benefits. Therefore, from the research documented in this review, it can be concluded that processed pulses and pulse-based foods can supply not only nutritive compounds, but also significant amounts of bioactive compounds, such as galactosides, phytates, protease inhibitors and phenolic compounds, with the potential to contribute to human health and wellbeing. Notably, the health benefits reported for legumes include anticarcinogenic and antihypertensive effects, and improvements in cardiovascular disease, diabetes type 2 and obesity could be related to a synergistic combination of the bioactive components present in legume seeds. With the increasing innovations in food-processing technology, the use of pulses in the development of new food products can meet consumers’ requirements for more nutritious and healthier products. Finally, it is interesting not only to know the phytochemical content of the processed legumes, but it also is necessary to know the amount of these compounds available in the gut once they are absorbed to exert their beneficial effect. In the near future, it will be necessary to carry out in vitro digestibility studies to determine the amount that reach the gut and are available to exert their healthy effect.

## Figures and Tables

**Table 1:** Content of different bioactive compounds (α-galactosides, inositol phosphates, protease inhibitors, phenolic compounds and antioxidant activity) in autoclaved legumes.

Legumes	Autoclaving Conditions	α-Galactosides	Inositol Phosphates	Protease Inhibitors	Phenolic Compounds	Antioxidant Activity	References
Adzuki bean	121 °C; 15 min			0.10 TIU/mg ↑			[53]
Pre-soaked bean (two vars.)	116 °C; 42 min	30.2–24.25 mg/g ↓	10.59–10.53 mg/g ↓	0.47–0,43 TIU/mg ↓	Total phenols: 9.65–11.80 µg/g ↓	ORAC: 16.03–16.03 µmol TE/g ↓	[5]
Pre-soaked pinto bean	116 °C;10–30 min	46.9–57.6 g/100 g ↑					[54]
Unsoaked kidney bean (three vars.)	121 °C; 30 min	0.55–0.65 g/100 g ↓	6.94–8.43 mg/g ↓	n.d. * ↓	Tannins: 1.51-14.37 mg/g ↓		[55]
Pre-soaked kidney bean (three vars.)	121 °C; 30 min	0.36–0.49 g/100 g ↓	6.59–8.90 mg/g ↓	n.d. ↓	Tannins: 1.34–12.91 mg/g ↓		[55]
Wild and cultivated Mexican bean	20 min	60.4 mg/g (mean) ↓	8.7 mg/g (mean) ↓				[56]
Brazilian bean (eight vars.)	121 °C; 15 min			0.48–1.00 TIU/mg ↓			[57]
Pre-soaked Brazilian bean (five vars.)	121 °C; 15 min			n.d. ↓			[57]
Pre-soaked kidney, pinto, black and borlotti bean	115 °C; 20 min				Total phenols: 0.38–0.94 mg GAE/100 g =	DPPH: 2.29–5.20 µmolTE/g = ABTS: 0.41–1.56 µmol TE/g = FRAP: 4.24–7.91 µmol TE/g ↓	[58]
Bean (two vars.)	121 °C; 7–12 min				Total phenols: 77.1–78.6 mg GAE/100 g ↓		[59]
Pre-soaked chickpeas	121 °C; 35 min	2.27 g/100 g ↓	0.71 g/100 g ↓	0.44 TIU/mg protein ↓	Tannins: 2.42 mg/g ↓		[47]
Whole chickpea (five cvs.)	120 °C; 60 min		112–335 mg/100 g ↓				[60]
Dehulled chickpea (five cvs.)	120 °C; 60 min		105–241 mg/100 g ↓				[60]
Chickpea	121 °C; 15 min			0.10 TIU/mg ↑			[53]
*Dolichos lablab* (Vulgaris var)	121 °C; 15–45 min		452–482 mg/100 g ↓	0.85–2.36 g/100 g ↓	Tannins: 0.05–0.13 g/100 g ↓ Total free phenols: 0.22–0.98 g/100 g ↓		[61]
Pre-soaked faba bean	120 °C; 30 min	1.43 g/100 g ↓	0.23 g/100 g ↓	0.35 TIU/mg ↓	Tannins: 0.58 g/100 mg ↓		[48]
Unsoaked faba bean (white and green)	121 °C; 15 min		8.90–9.27 mg/g ↑	0.46–1.23 mg/g ↓	Tannins: 4.72–6.51 mg/g ↑ in green; ↓ in white		[62]
Pre-soaked faba bean (white and green)	121 °C; 15 min		5.12–9.21 mg/g ↓ in green; ↑ in white	0.74–1.86 mg/g ↓	Tannins: 2.21–4.02 mg/g ↓		[62]
Pre-soaked faba bean (three genotypes)	115 °C; 20 min				Total phenols: 0.7–1.9 mg GAE/100 g ↓	DPPH: 4.6–9.9 µmol TE/g ↑ TEAC: 8.35–11.25 µmol TE/g ↓ ORAC: 20.54–33.25µmol TE/g ↓	[63]
Hyacinth bean	121 °C; 15 min			0.23 TIU/mg ↑			[53]
Pre-soaked lentil	121 °C; 35 min	1.42 g/100 g ↓	2.4 g/ g ↓	0.19 TIU/mg ↓	Tannins: 0.82 g/100 g ↓		[64]
Lentil (two vars.)	121 °C; 7 min				Total phenols: 318.3–533.4 mg GAE/100 g ↓		[65]
Lima bean	121 °C; 10–20 min		8.89 mg/ g ↓	n.d. ↓	Tannins: 0.23–0.32 g/100 g ↓		[66]
Black-eyed pea	121 °C; 15 min			0.22 TIU/mg ↑			[53]
Pea (two vars.)	121 °C; 12 min				Total phenols: 80.0–162.1 mg GAE/100 g ↓		[59]
Pigeon pea	121 °C;15 min			0.41 TIU/mg ↑			[53]
Mung bean	121 °C; 15 min			0.11 TIU/mg ↑			[53]
Soybean	121 °C; 15 min			0.09 TIU/mg ↑			[53]
Pre-soaked soybean	115 °C; 20 min				Total phenols: 0.71 mg GAE/100 g ↓	DPPH: 0.91µmol TE/g ↓ ABTS: 0.27 µmol TE/g ↓ FRAP: 2.18 µmol TE/g ↓	[58]

* n.d. not detected; TIU: trypsin inhibitor units; GAE: gallic acid equivalents; ORAC: oxygen radical absorbance capacity assay; DPPH: 2,2-diphenyl-1- picrylhydrazyl assay; TEAC: Trolox equivalent antioxidant capacity assay; FRAP: ferric reducing antioxidant power assay; ABTS: free radical scavenging ability assay; TE: Trolox equivalents; ↑: increase; ↓: decrease; =: no change.

**Table 2:** Content of different bioactive compounds (α-galactosides, inositol phosphates, protease inhibitors, phenolic compounds and antioxidant activity) in extruded legumes.

Legumes	Extrusion Conditions	α-Galactosides	Inositol Phosphates	Protease Inhibitors	Phenolic Compounds	Antioxidant Activity	References
Kidney bean (unsoaked)	140 °C and 180 °C; 18% and 22% moisture		9.64–10.90 mg/g ↓	n.d. *	Tannins: 196–223 mg/100 g ↓ Total phenols: 539–621 mg/100 g ↓		[104]
Kidney bean (pre-soaked)	140 °C and 180 °C; 18% and 22% moisture		9.53–10.41 mg/g ↑	n.d.	Tannins: 171–190 mg/100 g ↓ Total phenols: 413–494 mg/100 g ↓		[104]
Bean (four varieties)	120–140 °C; 25–30% moisture	27.75–36.20 mg/g ↑					[65]
Kidney bean var. Pinto	150–155 °C; 20% moisture	37.7 mg/g ↓	4.71 mg/g ↓		Tannins:2.75 g eq cat/kg ↓		[106]
Pinto bean	110 °C to 163 °C. 18.8 to 28.95% moisture	33.3–47.8 mg/g ↑					[107]
Navy and pinto bean (pre-soaked)	85 °C 36% moisture	30.45–33.65 mg/g ↓					[108]
Bean (three cultivars)	120 °C-180 °C. 14%-20% moisture				Total Phenols: 24.12–102.68 mg/100 g	TEAC: 69.26–77.88 µM Trolox/g ↓	[109]
*Canavalia* *ensiformis*	155 °C; 20% moisture			1.40 TIU/mg			[110]
Chickpea (unsoaked)	140 °C and 180 °C; 18% and 22% moisture		7.33–8.16 mg/g ↓	n.d.	Tannins: 190–245 mg/100 g ↓ Total phenols: 190–245 mg/100 g ↓		[104]
Chickpea (pre-soaked)	140 °C and 180 °C; 18% and 22% moisture		7.35–8.00 mg/g ↓ =	n.d.	Tannins: 195–214 mg/100 g ↑ Total phenols: 270–380 mg/100 g ↓		[104]
Chickpea	160 °C; 17% moisture	26.39 mg/g ↓					[111]
Chickpea (deffated)	130 °C; 14% moisture		12.6 µmol/g ↓		Total Phenols: 48.7 mg/100 mg ↑		[112]
Chickpea (germinated and dehulled)	180 °C; 16% moisture		1.33 mg/g ↓		Total phenols: 7.35 mg GAE/g ↑	DPPH: 45.46% ↑	[113]
Faba bean (unsoaked)	140 °C and 180 °C; 18% and 22% moisture		6.05–6.86 mg/g ↑	n.d.	Tannins: 397–438 mg/100 g ↓ Total phenols: 635–750 mg/100 g ↓		[104]
Faba bean (pre-soaked)	140 °C and 180 °C; 18% and 22% moisture		4.80–5.00 mg/g ↑	n.d.	Tannins: 362–426 mg/100 g Total phenols: 618–644 mg/100 g ↓		[104]
Lentil	160 °C 17% moisture	14.02 mg/g ↓					[111]
Split lentils	140 °C-160 °C-180 °C; 14%-18%-22% moisture		0.08–0.57 mg/g ↓	0.013–0.049 TIU/mg ↓	Tannins: 0.011–0.065 mg CE/100 g ↓ Total phenols 2.4–5.1 mg GAE/g ↓		[114]
Pea cv Ballet	145 °C-25% moisture	46.9 mg/g ↑	11.23 mg/g ↓	0.34 TIU/g ↓	Tannins: 0.02 g CE/kg ↓ Total Phenols: 0.23 g/kg ↓		[106]
Pea (unsoaked)	140 °C and 180 °C; 18% and 22% moisture		7.90–8.34 mg/g ↓	n.d.	Tannins: 236–278 mg/100 g ↓ Total phenols: 392–430 mg/100 g ↓		[104]
Pea (pre-soaked)	140 °C and 180 °C; 18% and 22% moisture		7.14–7.60 mg/g↓	n.d.	Tannins. 200–233 mg/100 g ↓ Total phenols: 343–379 mg/100 g ↓		[96]
Pea	160 °C 17% moisture	23.45 mg/g ↓					[111]
Pea	150–155 °C; 20% moisture	46.9 mg/g =	4.10 mg/g ↓		Tannins: 0.02 g CE/kg ↓		[106]

* n.d. not detected; TIU: trypsin inhibitor units; CIU: chymotrypsin inhibitor units; CE: caechin equivalents; GAE: gallic acid equivalents; DPPH: 2,2-diphenyl-1- picrylhydrazyl assay; TEAC: Trolox equivalent antioxidant capacity assay; ↑: increase; ↓: decrease; =: no change.

**Table 3:** Bioactive compounds (α-galactosides, inositol phosphates, protease inhibitors, phenolic compounds and antioxidant activity) content in extruded legume mixtures with other materials.

Legume Mixtures	Extrusion Conditions	α-Galactosides	Inositol Phosphates	Protease Inhibitors	Phenolic Compounds	Antioxidant Activity	References
Bean/rice (30/70)	80 °C; 14% moisture		16.59 mg/g		Tannins: 7.57 mg/g		[14]
Red common bean (15, 30 and 45%)/corn starch	160 °C; 22% moisture		0.54–2.33 mg/g ↓	n.d. *	40.94–94.82 mg FAE/100 g ↓	DPPH: 213.93–642.33 µmol TE/100 g ↓ ORAC: 388.69–1527.27 µmol TE/100 g ↓	[37]
Navy common bean (15. 30 and 45%)/corn starch	160 °C; 22% moisture		0.56–2.19 mg/g ↓	n.d.	28.27–45.96 mg FAE/100 g ↓	DPPH: 41.42–126.23 µmol TE/100 g ↓ ORAC: 254.36–584.46 µmol TE/100 g ↓	[37]
Bean/carob/rice (different formulas: 20–40%/0–10%/50–80%)	125 °C; 20% moisture	19.73–34.30 mg/g ↑	3.65–6.11 mg/.g ↓	n.d.	Total phenols: 0.92–3.25 mg CE/g ↓	ORAC: 8.92–11.89 µmol Trolox/g ↑	[100]
Kidney bean/corn starch (80/20)	150 °C. 20% moisture	45.63 mg/g ↓	7.08 mg/g ↓	0.25 TIU/mg ↓			[115]
Black bean with (0–4%) sodium bicarbonate	160 °C; 20% moisture	15.2–22.5 mg/g ↑					[116]
Bean/corn (60/40)	from 150 °C to 190 °C; from 14.5% to 18.0% moisture				Phenols: 6.46–17.40 mg GAE/g	CUPRAC: 9.55–37.02 µM Trolox eq/g; β-carotene 8.94–34.20%	[117]
Chickpea mixed with starch and fiber	150 °C. 20% moisture	14.38 mg/g ↓					[111]
Chickpea/corn starch (80/20)	150 °C. 20% moisture	44.21 mg/g ↓	5.62 mg/g ↑	2.52 TIU/mg ↓			[115]
Chickpea (germinated and dehulled) (10–30%)/corn/tomato pomace (5%)	180 °C; 16% moisture		1.20–1.00 mg/g ↓	n.d.	9.37–11.11 mg GAE/g ↑	DPPH: 53.82–56.33% ↑	[113]
Fermented chickpea/yogurt/locus bean gum	140 °C and 150 °C; 17% moisture		6.12–10.38 mg/g↓	2.02–8.82 TIU/mg ↓	Tannins: 0.37–1.03 mg CE/g ↓		[118]
Faba bean/corn starch (80/20)	150 °C; 20% moisture	31.66 mg/g ↓	6.25 mg/g ↓	0.44 TIU/mg ↓			[115]
Lentil mixed with nutritional yeast	160 °C; 17% moisture	33.35–49.55 mg/g ↑	2.72–3.94 mg/g ↓	0.20–0.28 TIU/mg ↓			[97]
Lentil mixed with starch and fiber	150 °C. 20% moisture	12.82 mg/g ↓					[111]
Red lentil (dehulled)/fiber (wheat, apple, nutriose^®^) (4 different formulations with at least 68% lentil)	160 °C; 17% moisture	24.60–42.52 mg/g ↑	1.38–4.62 mg/g ↓	n.d	4.51–9.38 mg GAE/g ↑	DPPH 6.63–63.56 EC_50_ mg/mL ↓ β-carotene assay 2.66–9.75 EC50 mg/mL ↑ ↓ TBARS: 1.52–4.59 EC_50_ mg/mL ↓	[119,120]
Lupin/corn starch (80/20)	150 °C; 20% moisture	83.76 mg/g ↓	5.99 mg/g ↑	0.29 TIU/mg ↓			[115]
Pea/carob/rice (different formulas: 20–40%/0–10%/50–80%)	125 °C; 20% moisture	24.80–50.21 mg/g ↑	3.14–4.60 mg/g ↓	n.d*.	Total phenols: 2.19–5.55 mg CE/g ↓	ORAC:9.81–12.00 µmol Trolox/g ↑	[18]
Pea mixed with starch and fiber	150 °C. 20% moisture	38.7 mg/g ↑					[111]
Pea/corn starch (80/20)	150 °C. 20% moisture	74.63 mg/g ↑	7.86 mg/g ↑	0.24 TIU/mg ↓			[115]

* n.d. not detected; TIU: trypsin inhibitor units; GAE: gallic acid equivalents; eq cat: catechin equivalents; DPPH: 2,2-diphenyl-1- picrylhydrazyl assay; ORAC: oxygen radical absorbance capacity assay; CUPRAC: cupric reducing antioxidant capacity *assay;* TBARS: thiobarbituric acid reactive substance assay; TE: Trolox equivalents; ↑: increase; ↓: decrease.

**Table 4:** Content of different bioactive compounds (α-galactosides, inositol phosphates, protease inhibitors, phenolic compounds and antioxidant activity) in uncooked legume-based pasta.

Legumes	α-Galactosides	Inositol Phosphates	Protease Inhibitors	Phenolic Compounds	Antioxidant Activity	References
100% legume (faba, lentil or black-gram flours)	44–57 mg/g	14.5–18 IP6 mg/g	7.8–11.3 TIU/mg			[155]
Commercial samples manufactured with lentil, bean and pea				Total phenols: 632–1743 mg GAE./100 g		[156]
Bean and carob fruit/rice	10–45 mg/g	3–9 mg/g IP total 2–5 mg/g IP6	2–13 TIU/mg 2–17 CIU/mg	Total phenols: 2.9–7.3 mg CE/g	ORAC: 5.3–13.7 µmol Trolox/g	[157]
Hard-to-cook bean protein hydrolysate/wheat semolina					ABTS: 15–31 mM/mg	[146]
Lima bean and cowpea/wheat					TAC: 26.09–31.84 mg Trolox eq./g	[158]
Faba bean/wheat				Total phenols: 185 mg GAE/100 g	ORAC: 1017 mg Trolox eq./100 g	[159]
Germinated and fermented cowpea flour/wheat semolina		677 mg IP6/g			TAC:31.9 µmol Trolox eq./g	[160]
Lentil				Total phenols: 1.42–2.14 mg GAE/g		[131]
Pea			0.45–1.09 TIU/mg			[151]
Pea flour, red lentil flour or 60% grass pea and 40% chickpea flour				Total phenols: 87–176 mg GAE/100 g	ORAC: 1851–3789 mg Trolox eq./100 g	[161]

CE: catechin equivalents; GAE: gallic acid equivalents; TAC: total antioxidant capacity; ORAC: oxygen radical absorbance capacity assay; ABTS: free radical scavenging ability assay.

**Table 5:** Content of different bioactive compounds (α-galactosides, inositol phosphates, protease inhibitors, phenolic compounds and antioxidant activity) in cooked legume-based pasta.

Legumes	α-Galactosides	Inositol Phosphates	Protease Inhibitors	Phenolic Compounds	Antioxidant Activity	References
100% legume (faba, lentil or black-gram flours)	12–34 mg/g ↓	12.8–15 IP6 mg/g ↓	1.52–2.5 TIU/mg ↓			[155]
Commercial samples manufactured with lentil, bean and pea				Total phenols: 323–814 mg GAE/100 g ↓		[156]
Bean and carob fruit/rice	4–27 mg/g ↓	3–8 mg/g IP total = 2–5 mg/g IP6 =	2–17 TIU/mg ↓ 3–17 CIU/mg ↑	Total phenols: 2.3–5.8 mg CE/g ↓	ORAC: 5.1–10.3 µmol Trolox/g ↓	[157]
Bean/semolina				Total phenols: 6.45–9.68 mg CE/g		[162]
Lima bean and cowpea/wheat					TAC: 16.63–21.39 mg Trolox eq./g ↓	[158]
Hard-to-cook bean protein hydrolysate/wheat semolina					ABTS: 6.6.-7.9 mM/mg ↓	[146]
Carob fiber/wheat semolina				Total phenols: 3.21–4.8 mg GAE/g		[163]
Carob pod/wheat semolina				Total phenols: 5.3–12.2 mg GAE/g	ABTS: 0.25–1.35 mg Trolox eq./g FRAP: 2.05–6.9 mg Trolox eq./g	[164]
Chickpea/soya/different cereals		1050–9640 mg IP6/Kg				[138]
Germinated and fermented cowpea flour/wheat semolina		574 mg IP6/g ↓			TAC: 27.5 µmol Trolox eq./g	[154]
Lentil				Total phenols: 0.93–1.42 mg GAE/g ↓		[131]
Pea			0.23–0.29 TIU/mg ↓			[151]
Fermented pigeon pea/wheat semolina	n.d. *	0.24 g/100 g	n.d.	Tannins: 0.28 g GAE/100 g	TAC: 3.35 µmol Trolox eq./g	[153]
Germinated pigeon pea/wheat semolina		0.27 g/100 g	1.57 TIU/mg	Tannins: 0.19 g GAE/100 g	TAC: 5.8 µmol Trolox eq./g	[160]

* n.d.: not detected; GAE: gallic acid equivalents; CE: catechin equivalents; TAC: total antioxidant capacity; ORAC: oxygen radical absorbance capacity assay; FRAP: ferric reducing antioxidant power assay; ABTS: free radical scavenging ability assay; ↑: increase; ↓: decrease; =: no change.

## Supplementary Materials

The following are available online at https://www.mdpi.com/2304-8158/10/2/379/s1, Figure S1. A PRISMA flow chart for this manuscript.
