# Supplementary material for: Autoclaved and Extruded Legumes as a Source of Bioactive Phytochemicals: A Review
Source: Foods. 2021 Feb 9;10(2):379. doi: 10.3390/foods10020379 (PMC7919342; doi:10.3390/foods10020379)

**Figure S1.** PRISMA flow diagram for 'autoclaved and extruded legumes as a source of bioactive phytochemicals: a review' manuscript. Diagram from: Moher D, Liberati A, Tetzlaff J, Altman DG, The PRISMA Group (2009). Preferred Reporting Items for Systematic Reviews and Meta-Analyses: The PRISMA Statement. PLoS Med 6(7): e1000097. doi:10.1371/journal.pmed1000097.

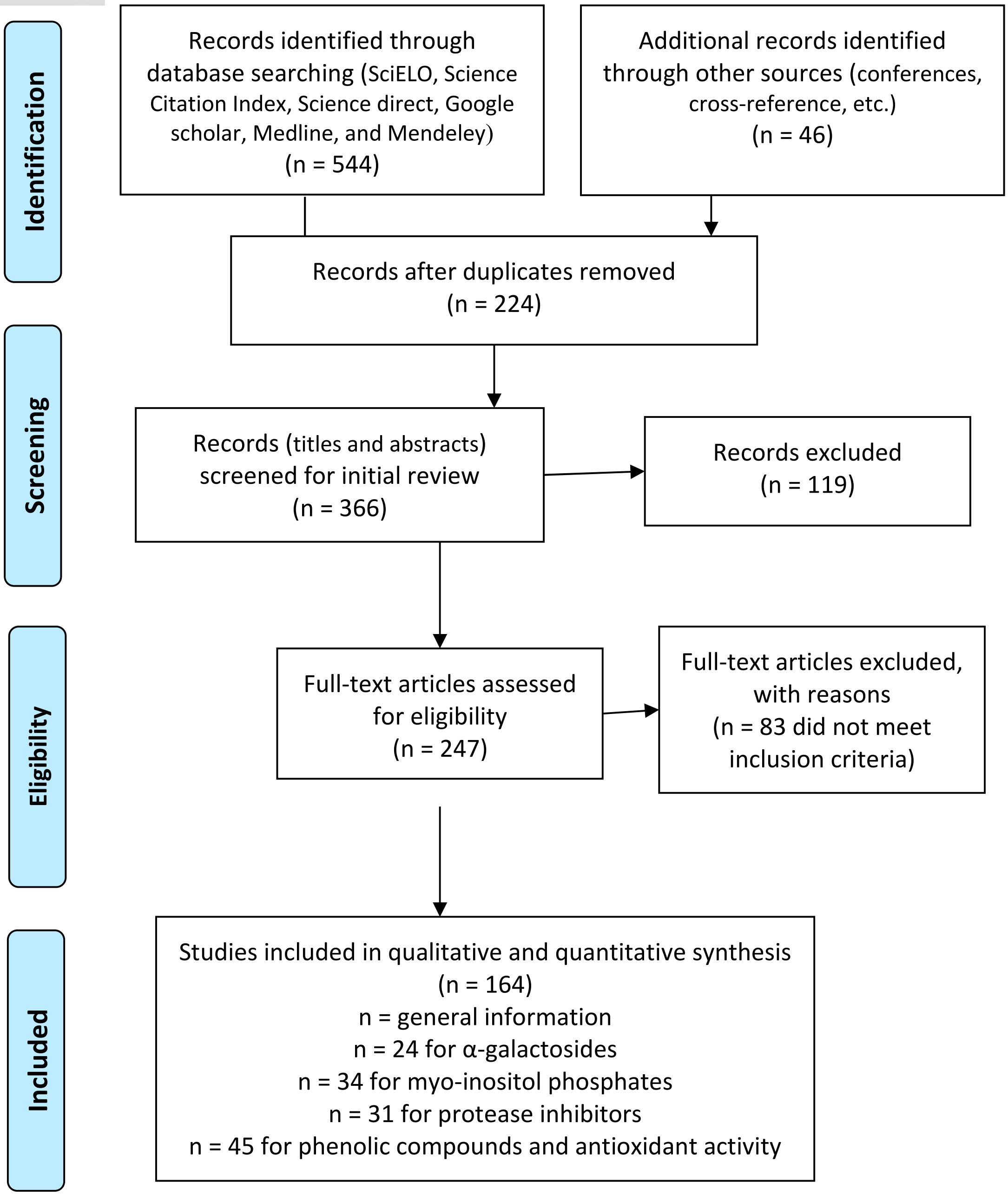

Supplement: Supplementary file 1 [file foods-10-00379-s001.pdf]
